# Dynamics of synthetic yeast chromosome evolution shaped by hierarchical chromatin organization

**DOI:** 10.1093/nsr/nwad073

**Published:** 2023-03-17

**Authors:** Sijie Zhou, Yi Wu, Yu Zhao, Zhen Zhang, Limin Jiang, Lin Liu, Yan Zhang, Jijun Tang, Ying-Jin Yuan

**Affiliations:** Frontiers Science Center for Synthetic Biology (Ministry of Education), Tianjin University, Tianjin 300072, China; Key Laboratory of Systems Bioengineering (Ministry of Education), School of Chemical Engineering and Technology, Tianjin University, Tianjin 300072, China; Frontiers Science Center for Synthetic Biology (Ministry of Education), Tianjin University, Tianjin 300072, China; Key Laboratory of Systems Bioengineering (Ministry of Education), School of Chemical Engineering and Technology, Tianjin University, Tianjin 300072, China; Institute for Systems Genetics, NYU Langone Health, New York, NY 10016, USA; Frontiers Science Center for Synthetic Biology (Ministry of Education), Tianjin University, Tianjin 300072, China; Key Laboratory of Systems Bioengineering (Ministry of Education), School of Chemical Engineering and Technology, Tianjin University, Tianjin 300072, China; School of Computer Science and Technology, College of Intelligence and Computing, Tianjin University, Tianjin 300350, China; Epigenetic Group, Frasergen Bioinformatics Co., Ltd., Wuhan 430000, China; Frontiers Science Center for Synthetic Biology (Ministry of Education), Tianjin University, Tianjin 300072, China; School of Computer Science and Technology, College of Intelligence and Computing, Tianjin University, Tianjin 300350, China; Department of Computer Science, University of South Carolina, Columbia, SC 29208, USA; Frontiers Science Center for Synthetic Biology (Ministry of Education), Tianjin University, Tianjin 300072, China; Key Laboratory of Systems Bioengineering (Ministry of Education), School of Chemical Engineering and Technology, Tianjin University, Tianjin 300072, China

**Keywords:** synthetic biology, genome rearrangement, hierarchical chromatin organization, synthetic yeast genome, SCRaMbLE, *Saccharomyces cerevisiae*

## Abstract

Synthetic genome evolution provides a dynamic approach for systematically and straightforwardly exploring evolutionary processes. Synthetic Chromosome Rearrangement and Modification by LoxP-mediated Evolution (SCRaMbLE) is an evolutionary system intrinsic to the synthetic yeast genome that can rapidly drive structural variations. Here, we detect over 260 000 rearrangement events after the SCRaMbLEing of a yeast strain harboring 5.5 synthetic yeast chromosomes (synII, synIII, synV, circular synVI, synIXR and synX). Remarkably, we find that the rearrangement events exhibit a specific landscape of frequency. We further reveal that the landscape is shaped by the combined effects of chromatin accessibility and spatial contact probability. The rearrangements tend to occur in 3D spatially proximal and chromatin-accessible regions. The enormous numbers of rearrangements mediated by SCRaMbLE provide a driving force to potentiate directed genome evolution, and the investigation of the rearrangement landscape offers mechanistic insights into the dynamics of genome evolution.

## Teaser

A specific landscape of over 260,000 rearrangements in SCRaMbLEd yeast harboring 5.5 synthetic chromosomes was uncovered and found to be a result of combinatorial effects of chromatin accessibility and 3D spatial contact probability.

## INTRODUCTION

Studying the processes and mechanisms of genome evolution is critical to understanding genetics and species diversity at the genomic level [1–4]. *Saccharomyces cerevisiae*, a powerful model organism for studying eukaryotic genome evolution [5–7], has been subjected to comparative genomic studies that have provided mechanistic insights [8–12]. These studies relied on relatively static genomic sequences and may have missed many details of process dynamics. Synthetic genomes, assembled from scratch and incorporated with a variety of designer features, have greatly facilitated the study of genome evolution and engineering, such as genome minimization [13], genetic codon recoding [14,15], the introduction of synthetic components [16] and data storage [17,18]. Synthetic Chromosome Rearrangement and Modification by LoxP-mediated Evolution (SCRaMbLE), with symmetrical loxP sites (loxPsym) positioned in the 3′ untranslated regions (3′ UTRs) of almost all non-essential genes in the Synthetic Yeast Genome Project (Sc2.0), has also recently been used in the study of genome evolution [16,19]. Induced Cre recombinase activity quickly triggers recombination between loxPsym sites and generates structural variations, including deletions, inversions, duplications and translocations [20–27].

In this study, we consolidated 5.5 synthetic chromosomes (synII, synIII, synV, circular synVI, synIXR and synX) in a single haploid strain with an orthogonal site-specific recombination system, which enabled the eliminations of counterpart native chromosomes [19,28–32]. In this strain with nearly a quarter of a synthetic genome, we induced SCRaMbLE and generated massive rearrangement events involving both intra- and inter-chromosomal recombination. We comprehensively sequenced this SCRaMbLEd pool and detected over 260 000 rearrangement events via a loxPsym junction analysis method. By analyzing each rearrangement event, we revealed a stable rearrangement landscape among synthetic chromosomes that was correlated with local chromatin structures and the three-dimensional genome architecture revealed via assay for transposase-accessible chromatin sequencing (ATAC-seq) and genome-wide chromosome conformation capture (Hi-C). Our study provides insight into the combinatorial effect of hierarchical chromatin organization on the dynamics of genome evolution.

## RESULTS

### Consolidation and SCRaMbLE of six synthetic chromosomes

**Figure 1.**
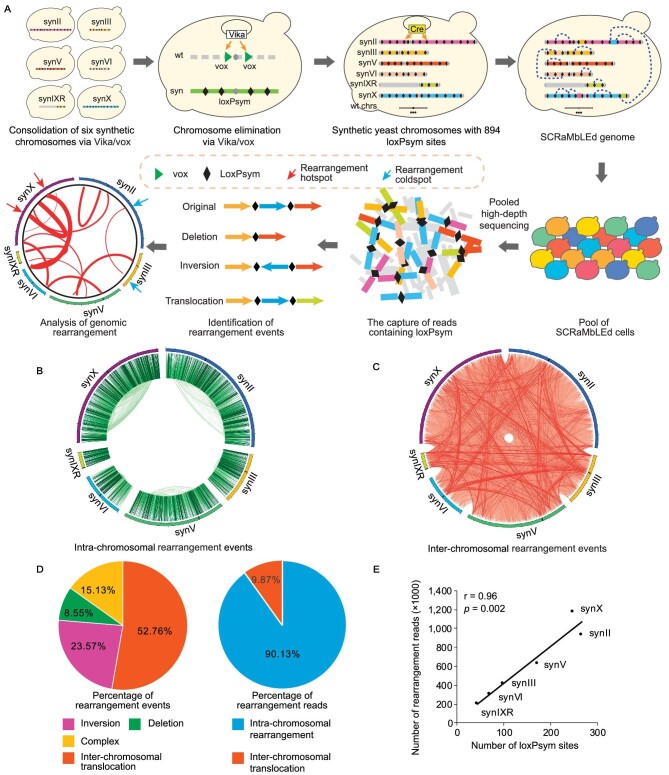
Consolidation and SCRaMbLE of six synthetic chromosomes. (A) Schematic diagram showing the construction of the synthetic yeast strain yZSJ025 and the analysis of rearrangement events following SCRaMbLE. (B) Circos plot of intra-chromosomal rearrangement events in yZSJ025. (C) Circos plot of inter-chromosomal rearrangement events in yZSJ025. Rearrangement events between two loxPsym sites are connected with a line and the color intensity represents the frequency of the event. (D) Classifications of rearrangement events with percentages of different groups calculated by event numbers and read numbers respectively. Different groups of events are labeled in different colors as indicated. (E) Correlation analyses between the rearrangement frequency and the number of loxPsym sites on each chromosome. Pearson correlation analysis was applied to determine the correlation coefficient and associated *P* values. SynVI is a ring chromosome.

To build the strain with multiple synthetic chromosomes, we used a stepwise method, starting with two haploid strains of opposite mating types, yYW169 (synV, synX) [33] and yZY192 (synII, synIII, synVI and synIXR) [16]. To accelerate consolidation, we developed a new strategy using chromosome elimination by Vika/vox, a site-specific recombination system orthogonal to Cre/loxP (Fig. 1A) [34,35]. First, we built a heterozygous diploid strain yYW268 by mating yYW169 and yZY192 (Fig. S1A). The centromeres of native chromosomes (II, III, V, VI, IX and X) were successively excised by Vika/vox recombination, leading to elimination of the chromosomes (Fig. S1B). We then obtained the yYW393 strain, whose ploidy was further tested by Next-Generation Sequencing (NGS) and flow cytometry. We confirmed that yYW393 was a diploid strain (Figs S2 and S3), indicating that endoreduplication of the counterpart synthetic chromosomes occurred after targeted elimination of native chromosomes by Vika/vox. We characterized the growth phenotypes of yYW393 and found that the yYW393 strain grew slightly slower on rich medium at a low temperature (25°C) (Fig. S4). Following sporulation and tetrad dissection, one haploid strain (yYW394), in which the six aforementioned native chromosomes were replaced with synthetic chromosomes, was obtained, comprising ∼2.61 Mb (∼22.0% of the yeast genome) (Table S1). Its karyotype and genome sequence were confirmed by NGS, flow cytometry and pulsed-field gel electrophoresis (Figs S2, S3, S5B and Table 1). The yYW394 strain grew robustly at 30°C but exhibited a growth defect at 37°C (Fig. S5C). To recover its fitness to wild-type levels, the adaptive laboratory evolution of yYW394 was performed, generating an evolved strain yZSJ025, which was used for further analysis (Fig. S5). We then performed spotting assays for yZSJ025 under 30 various conditions. Although growth of yZSJ025 on the Yeast Peptone Dextrose (YPD) agar plate at 37ºC has been largely recovered, it is obviously still slower-growing under some other tested conditions (Fig. S6). We speculated that the removal of tRNA genes from their native loci may be the primary reason for the growth defects. A recent investigation revealed that addition of a tRNA array can recover growth defects of a synthetic yeast strain [36].

**Table 1.** Overview of sequencing data.

Sample name	BioSample	Accession number	Type
yZY192	SAMN31499054	PRJNA895231	Genome_sequencing
yYW393	SAMN31499055	PRJNA895231	Genome_sequencing
yYW394	SRX10191928	PRJNA705059	Genome_sequencing
yZSJ025	SRX10191929	PRJNA705059	Genome_sequencing
yZSJ364	SRX10191930	PRJNA705059	Genome_sequencing
yZSJ365	SRX10191931	PRJNA705059	Genome_sequencing
yZSJ400	SRX10191933	PRJNA705059	Genome_sequencing
yZSJ490	SRX10191932	PRJNA705059	Genome_sequencing
yZSJ025-ATACseq-rep1	SAMN18129009	GSE168182	ATAC sequencing
yZSJ025-ATACseq-rep2	SAMN18129008	GSE168182	ATAC sequencing
yZSJ025-HiC-rep1	SAMN18129007	GSE168182	HiC sequencing
yZSJ025-HiC-rep2	SAMN18129006	GSE168182	HiC sequencing
yZSJ025		GSE219048	RNA sequencing

The evolved strain yZSJ025, comprising 894 loxPsym sites, was transformed with the Cre recombinase expression plasmid pYW085 (pRS413-pCLB2-Cre-EBD) (Fig. S7). SCRaMbLE was induced by the addition of β-estradiol. The SCRaMbLEd cells were then diluted in fresh YPD liquid medium without β-estradiol (Fig. 1A), and subjected to deep sequencing (∼600 000×). All reads (150 bp each) were screened for the presence of loxPsym sequences and then aligned to the reference sequence of the original synthetic chromosomes. Those with flanking sequences differing from the references, hereafter referred to as rearrangement reads, were then used to identify and classify rearrangement events [25] (Fig. S8). Identical reads were considered to represent a single rearrangement event. In total, 263 520 rearrangement events, including 124 499 (47.24%) intra- and 139 021 (52.76%) inter-chromosomal events, were detected (Figs 1B and C). We further analyzed the intra-chromosomal rearrangement events and found 62 106 (23.57%) inversions, 22 526 (8.55%) deletions and 39 867 (15.13%) complex rearrangement events, such as duplications and circularization events (Figs 1D and S8). We also counted the reads corresponding to each rearrangement. The numbers of identical reads represent the frequencies of corresponding rearrangement events. Inter-chromosomal recombination accounted for only 9.87% of the total read numbers, indicating that these are relatively low frequency events compared to intra-chromosomal rearrangements. We next investigated whether there is any chromosome preference in rearrangements. To answer this question, we plotted the number of rearrangements involving each chromosome against the number of loxPsym sites on that chromosome. As expected, the number of rearrangements was correlated with the number of loxPsym sites per chromosome (r = 0.96, *P* = 0.002) (Fig. 1E).

### A specific rearrangement pattern of the synthetic yeast chromosomes

**Figure 2.**
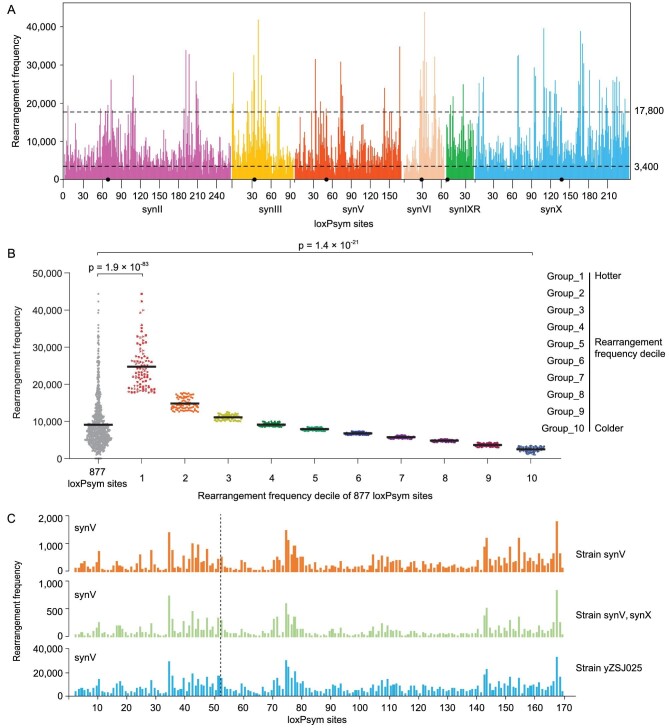
Investigating rearrangement frequencies revealed a specific rearrangement pattern among synthetic yeast chromosomes. (A) Landscape of rearrangement frequencies along synII, synIII, synV, synVI, synIXR and synX. Seventeen loxPsym sites were inserted in the synthetic telomere regions, where flanking sequences were not distinguishable from each other; these sites were excluded from the identification of rearrangement reads. Dark dots indicate centromeres. (B) Comparison of rearrangement frequencies at 877 loxPsym sites. These 877 loxPsym sites were divided into 10% steps according to rearrangement frequencies. The points represent the rearrangement frequencies at each loxPsym site. Horizontal lines indicate weighted means. *P* values were calculated using two-tailed paired two-sample *t*-tests. (C) Intra-chromosomal rearrangement patterns of synV following SCRaMbLE in three different yeast strains, yXZX846 (synV), yYW169 (synV, synX) and yZSJ025 respectively. Pearson correlation analysis was applied to determine the correlation coefficients and associated *P* values. The dotted line indicates centromeres. SynVI is a ring chromosome.

In theory, SCRaMbLE can generate rearrangements between any two loxPsym sites on synthetic chromosomes. However, different chromosomal loci showed high or low rearrangement frequencies (Figs 1B and C). To estimate the local rearrangement frequencies along the synthetic chromosomes in our SCRaMbLEd pool, we counted the number of rearrangement reads from each loxPsym site, generating an observable landscape of rearrangement frequencies (Fig. 2A). Seventeen loxPsym sites were inserted in synthetic telomere regions, where the flanking sequences were not distinguishable from each other; these sites were excluded from the identification of rearrangement reads. Notably, rearrangement reads were identified from all 877 loxPsym sites, indicating that the sequencing coverage was sufficient to include all loci. The loxPsym sites differed from each other in their rearrangement frequencies, ranging from 1027 to 44 353 per site. These 877 loxPsym sites were divided into 10% steps according to rearrangement frequencies (Fig. 2B). We defined 88 loxPsym sites with the highest rearrangement frequencies as hotspots and 88 loxPsym sites with the lowest rearrangement frequencies as coldspots. The cut-offs for hotspots and coldspots were >17 800 and <3400 rearrangement events per site respectively. These rearrangement frequencies were significantly different from the average frequency of all loxPsym sites (Fig. 2B). Interestingly, the rearrangement frequency landscapes of independent SCRaMbLE experiments were highly reproducible for all the synthetic chromosomes, indicating that certain areas of the genome have the potential to undergo rearrangement at higher frequencies (Figs S9 and S10).

We also compared the rearrangement patterns of the same synthetic chromosome in separate strains containing different numbers of synthetic chromosomes. Similar intra-chromosomal rearrangement patterns were observed for synV in the three synV-containing strains (yXZX846, yYW169 and yZSJ025) (Figs 2C and S11A–C). The same result was observed for synX in the two synX-containing strains (yYW169 and yZSJ025) (Figs S11D and E). Overall, our results demonstrate that the SCRaMbLE patterns followed the same biological reason on each synthetic chromosome with reproducible rearrangement hotspots and coldspots. In addition, we also carried out RNA-seq to investigate the correlation of gene transcription and adjacent recombination frequency of yZSJ025, which showed no obvious correlation, with an R value of 0.16 (Fig. S12).

### Rearrangement frequency correlates with chromatin accessibility

**Figure 3.**
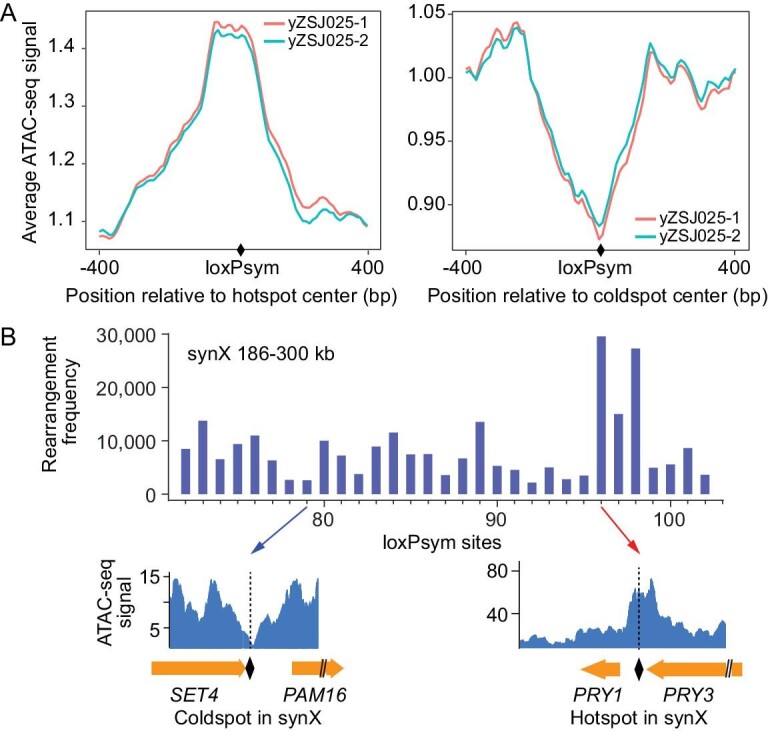
Correlation of rearrangement frequency with chromatin accessibility. (A) Average ATAC-seq signals of the hotspot- and coldspot-centered 800 bp regions. (B) ATAC-seq signals for a typical rearrangement hotspot and a coldspot in the 186–300 kb region of synX.

To determine whether the specific rearrangement patterns we observed were due to an underlying biological mechanism, we further investigated the genomic landscape. SCRaMbLE requires physical interaction between Cre recombinase and loxPsym sites, suggesting the importance of chromatin accessibility in determining the rearrangement frequency in SCRaMbLEd cells. To test this hypothesis, we measured genome-wide chromatin accessibility in the yZSJ025 strain by ATAC-seq [37]. The ATAC-seq signals from windows 400 bp upstream and downstream of each loxPsym site were collected and processed. The signals from each loxPsym site were normalized with the average signals of all 877 loxPsym sites. We analyzed the ATAC-seq signals of 877 loxPsym sites in 20% steps based on rearrangement frequencies. Interestingly, ATAC signals are well correlated with the rearrangement frequencies (Fig. S13). We further analyzed the defined rearrangement hotspots and coldspots. Statistical analysis showed that the average ATAC-seq signals of hotspots were significantly higher, while coldspot signals were significantly weaker (Fig. 3A). The rearrangement frequencies and ATAC-seq signals of a typical loxPsym hotspot and coldspot from synX are shown in Fig. 3B. The presented coldspot, the loxPsym site in the *SET4* 3’ UTR, showed a weak ATAC-seq signal, while as a hotspot, the loxPsym site in the *PRY3* 3′ UTR showed a strong ATAC-seq signal. In addition, we observed that nucleosome occupancy was relatively low in hotspots and high in coldspots (Fig. S14). Both ATAC-seq and nucleosome occupancy analyses indicated that chromatin accessibility is critical for determining the patterns of rearrangement frequencies.

We then aligned the hotspots and coldspots to the corresponding regions of native chromosomes. Using the same approach applied previously, we extracted and analyzed pre-published ATAC-seq data from wild-type yeast [37] (Fig. S15). ATAC-seq signals peaked at the positions of hotspots, and remained weak at coldspots on all six chromosomes, suggesting that sequence modifications of Sc2.0 do not perturb chromatin accessibility.

### Rearrangement frequency also correlates with 3D chromosome conformation

**Figure 4.**
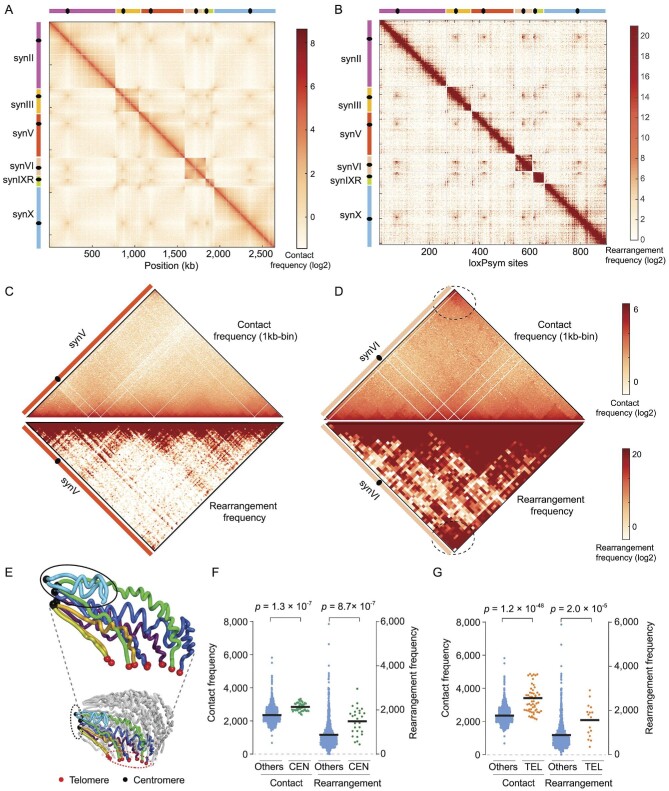

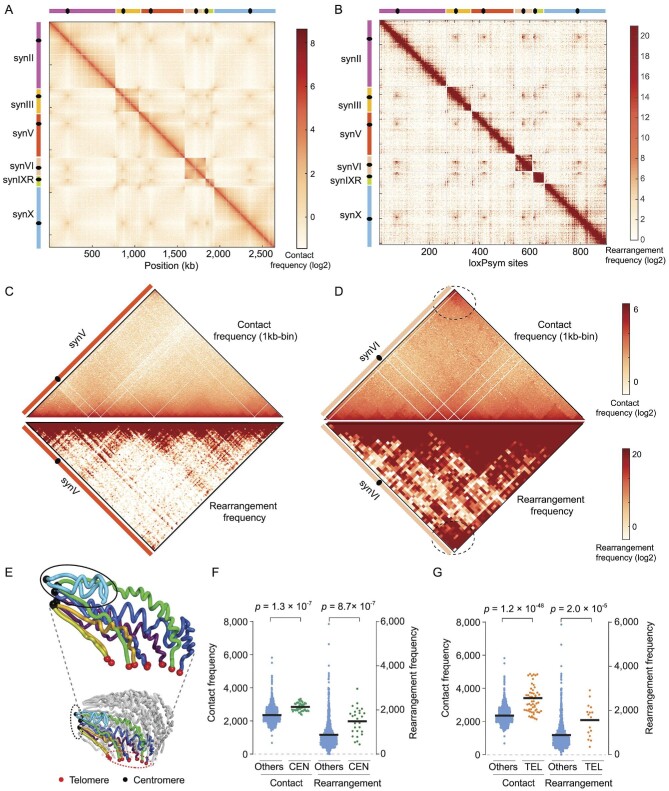
Correlation of the rearrangement frequency with 3D chromosome conformation. (A) Hi-C heatmap. The heatmap value for a site (*i, j*) is the contact probability (log2) between genomic loci *i* (horizontal axis) and *j* (vertical axis). Both axes are displayed at a 1 kb resolution. Spots with different heatmap values from low to high are colored from light yellow to red as indicated. (B) Rearrangement frequency heatmap. The heatmap value for a site (*i, j*) is the rearrangement frequency (log2) of the event occurring at loci *i* (vertical axis) and *j* (horizontal axis). Spots with different heatmap values are labeled in red with different intensities as indicated. (C) Hi-C heatmap and rearrangement frequency heatmap of synV. (D) Hi-C heatmap and rearrangement frequency heatmap of ring synVI. (E) 3D structures of synthetic chromosomes inferred from the Hi-C contact map displayed in panel (A). Synthetic chromosomes are labeled with different colors and the native chromosomes are shown in gray. Centromeres and telomeres are represented by dark and red spheres respectively. Dark and red dashed circles represent centromere and telomere regions respectively. (F) Comparisons of the contact probability and frequency of rearrangement events at loci in pericentromeric regions (CEN) and in other regions (Others). CENs are centromere-centered 10 kb regions. (G) Comparisons of the contact probability and frequency of rearrangement events at loci in peritelomeric regions (TEL) and in other regions (Others). TELs are ≤5 kb from telomeres. For (D) and (E), each data point represents a 1 kb bin in regions as indicated. Horizontal lines indicate weighted means. *P* values were calculated using two-tailed paired two-sample *t*-tests. SynVI is a ring chromosome.

Next, we aimed to explore whether the rearrangement frequency was correlated with the spatial proximity of loxPsym sites. Our SCRaMbLE system provided a unique platform for statistically evaluating the role of spatial proximity in chromosomal rearrangements. A Hi-C analysis was thus carried out for yZSJ025, generating a contact map with the frequencies of spatial contacts between any two genomic loci (Fig. S16). We extracted contact frequencies from the synthetic chromosomes (Fig. 4A), and plotted rearrangement frequencies in a similar heatmap to facilitate direct comparison (Fig. 4B). The regions near the diagonal represent proximal loci on each chromosome. Accordingly, these intra-chromosomal regions were the ‘hottest’ in the Hi-C map (Fig. 4A), and they also exhibited the highest rearrangement frequency (Fig. 4B). Rearrangements tended to occur most frequently between adjacent loxPsym sites for all synthetic chromosomes, as shown by the statistical analysis of all rearrangement reads (Fig. S17). Although inclusion of a circular chromosome in our system was not originally designed, it served as a great internal control and gave the opportunity to explore the difference between the rearrangement of the ring chromosome and that of other linear chromosomes in a cell. Compared to other linear chromosomes, the ring chromosome exhibited higher frequencies of SCRaMbLE events between two peritelomeric regions that are now adjacent to each other (Figs 4C and D, and S18). Indeed, a similar observation on a different yeast circular chromosome, synII, agreed with our conclusion that circular chromosomes exhibit stronger frequencies of SCRaMbLE events between two peritelomeric regions [38].

Notably, the inter-chromosomal contact probability in the regions of centromeres and telomeres was obviously higher than that in other regions (Fig. 4A), consistent with centromeres being clustered around the spindle pole body and telomeres being clustered with the nuclear envelope in both wild-type [39] and synthetic chromosomes [33] (Fig. 4E). The 3D conformations showed the circularity of synVI. Compared to other regions, pericentromeric regions exhibited a higher inter-chromosomal rearrangement frequency (Fig. 4B and F). Similar results were found for the peritelomeric regions of the synthetic chromosomes (Fig. 4B and G). Taken together, our results suggested that rearrangement events are generally more likely to occur between genomic loci in closer spatial proximity to each other.

### Combinatorial effects of chromatin accessibility and chromosome conformation

**Figure 5.**
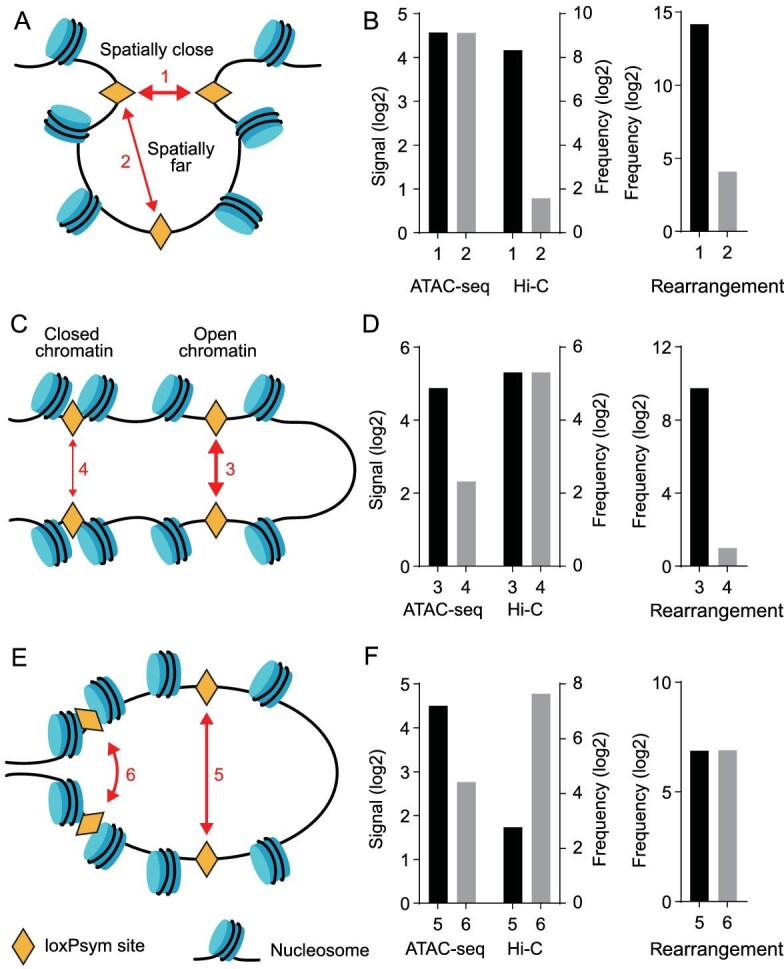
Mechanistic models of the effects of hierarchical chromatin organization on chromosomal rearrangement. (A) A schematic model interpreting the differences in the frequencies of two rearrangement events with different contact probabilities. (B) The loxPsym positions of pair 1: synX 183 kb and synX 184 kb; pair 2: synX 184 kb and synX 342 kb. (C) A schematic model interpreting the difference in the frequencies of two rearrangement events under differences in chromatin accessibility. (D) The loxPsym positions of pair 3: synII 186 kb and synII 204 kb; pair 4: synV 346 kb and synV 384 kb. (E) A schematic model interpreting two rearrangement events with similar frequencies as a result of counter-effects according to contact probability and chromatin accessibility. (F) The loxPsym positions of pair 5: synVI 125 kb and synVI 202 kb; pair 6: synII 504 kb and synII 507 kb.

As both chromatin accessibility and spatial proximity influence rearrangement frequency, we hypothesized that they have combinatorial effects on yeast chromosome organization. We tested our proposed model based on individual rearrangement events. The results indicated that if two loxPsym sites showed similar chromatin accessibility (Fig. 5A), the frequency of rearrangement events was determined by their spatial proximity (Fig. 5B). For loxPsym sites with similar spatial proximity (Fig. 5C), the rearrangement frequency was mainly affected by their chromatin accessibility (Fig. 5D). Most interestingly, if two loxPsym sites presented an open chromatin conformation but were spatially distant, they presented a similar recombination frequency to other pairs of loxPsym sites that were proximal but hidden within closed chromatin structures (Figs 5E and F). In other words, the effect of chromatin accessibility on the rearrangement frequency could be compensated by the difference in spatial proximity, and vice versa. More generally, the chromatin architecture and spatial conformation in the yeast genome affected the rearrangement frequency in a combinatorial manner during SCRaMbLE.

## DISCUSSION

In this study, we induced SCRaMbLE in a novel strain with multiple synthetic chromosomes. Over 260 000 rearrangement events were detected in the SCRaMbLEd pool, covering all 877 loxPsym sites. This finding revealed the tremendous plasticity of the yeast genome. Previous studies involving SCRaMbLE have mainly focused on the correlation of chromosomal rearrangement with nucleotide sequences [21,23,25,27,40]. The biochemical essence of SCRaMbLE is the recombination between pairs of different loxPsym sites on synthetic chromosomes catalyzed by Cre recombinase [41]. We speculate that local chromatin structure affects rearrangement through the accessibility of loxPsym sites to Cre and that 3D chromosome conformation affects the contact probability at any two loxPsym sites. In this study, we discovered that the rearrangement frequency landscape was indeed molded by chromatin structure and that the recombination hotspots tended to occur in 3D spatially proximal and chromatin-accessible regions. Similarly, it was observed in non-synthetic chromosomes that the frequency of double strand break (DSB), a main trigger of chromosomal recombination, is also associated with chromosome accessibility [42,43]. Large chromosome structural variations, including simple Structure Variantions (SVs) (e.g. deletions, duplications and inversions), chromosome fusion, copy-number alterations and other complex SVs, have been found to be associated with certain types of cancers [44,45]. Recently, researches focused on the occurrence of such variations that lead to cancers highlighted the importance of spatial contact probability within the 3D organization in this process [46,47].

The use of synthetic chromosomes and SCRaMbLE allowed us to statistically demonstrate that inter-chromosomal rearrangement hotspots strikingly clustered at the peritelomeric and pericentromeric regions. Our results are consistent with previous reports showing that increased inter-chromosomal reshuffling has occurred in the peritelomeric regions of domesticated and wild yeast isolates during genome evolution [8,11]. Peritelomeric regions are functionally enriched for genes involved in secondary metabolism and stress responses that contribute to environmental adaptation [48–51]. A high frequency of rearrangement in these regions could thus be an important driving force for evolution. However, others were surprisingly found located in the pericentromeric regions. Centromeric rearrangements may result in the formation of acentric or dicentric chromosomes, and thus lead to cell death, and could not be detected as rearrangement hotspots in natural living cells [52]. In addition, centromeric rearrangements result in the swapping of chromosomal arms between two chromosomes, which can cause reproductive isolation and promote incipient speciation [53–55]. Taken together, recombination events in the pericentromeric regions are rarely observed in nature, and sequences in these regions are believed to be relatively stable across species. Nevertheless, the sequencing data of our SCRaMbLEd pool may reveal the hidden fact that the pericentromeric regions of *S. cerevisiae* are prone to rearrange at the beginning, but the rearrangements are evolutionarily eliminated in the later adaptive process. Taking the combinatorial effect of chromatin accessibility and chromosome conformation into consideration, we speculated that chromatin structures might play an important role in genome evolution in terms of the effects on rearrangements in peritelomeric and pericentromeric regions.

Consolidation of all 16 yeast synthetic chromosomes and novel tRNA neochromosome in the final Sc2.0 strain is likely to happen soon [16,36,56]. The flexible and controllable synthetic yeast genome provides a unique model to systematically interrogate and explore the dynamics of eukaryotic genome evolution. The investigation of a large number of rearrangements indicated the tremendous plasticity of the yeast genome and the importance of hierarchical chromatin organization to the regional rates of chromosomal variation during genome evolution. These findings provide crucial insights into the variability and complexity of synthetic genome design. Additionally, the influence of chromatin organization needs to be considered in the design and engineering of higher organism genomes.

## Data Availability

Genome sequencing data have been submitted to the National Center for Biotechnology Information (NCBI) Sequence Read Archive (SRA) under accession numbers PRJNA705059 and PRJNA895231. The ATAC-seq and Hi-C sequencing data have been submitted to the NCBI Gene Expression Omnibus (GEO) under accession number GSE168182. The ATAC-seq data of wild-type *S. cerevisiae* are available from GEO under accession number GSE66386.

## Supplementary Material

nwad073_Supplemental_File
