# Supplementary material for: Dynamics of synthetic yeast chromosome evolution shaped by hierarchical chromatin organization
Source: Natl Sci Rev. 2023 Mar 17;10(5):nwad073. doi: 10.1093/nsr/nwad073 (PMC10202648; doi:10.1093/nsr/nwad073)
Supplement: nwad073_Supplemental_File [file nwad073_supplemental_file.pdf]

Dynamics of synthetic yeast chromosome evolution shaped by hierarchical chromatin organization

Supplementary Materials

Sijie Zhou†, Yi Wu†, Yu Zhao†, Zhen Zhang, Limin Jiang, Lin Liu, Yan Zhang, Jijun Tang, and Ying-Jin Yuan*

†These authors contributed equally.

*Correspondence: Ying-Jin Yuan, E-mail: yjyuan@tju.edu.cn

Supplementary materials include:

Supplementary Note

Figure S1-S18

Table S1-S6

Supplementary Note

Strains and plasmids

The plasmids and yeast strains used in this study were listed in Supplementary Table 4.

Synthetic chromosome sequences and versions

The haploid draft strain yYW394 contains multiple synthetic chromosomes of synII (chr02_9_03), synIII (chr03_9_02), synV (chr05_9_04), synVI (chr06_9_03), synIXR (genebank JN020955), and synX (chr10_9_01) as published before [1-6]. The final strain yZSJ025 contains the same synthetic chromosomes except some variants to improve its fitness, as listed in Supplementary Table 2. The genome sequencing data was submitted to NCBI Sequence Read Archive (SRA) under accession number PRJNA705059.

Plasmid construction

All plasmids were constructed using standard molecular cloning techniques and transformed into the *E. coli* TOP 10 strain following standard protocols. Plasmid constructs were verified by restriction digestion and Sanger sequencing by Genewiz. The applied restriction endonucleases and Phusion PCR kits were from New England BioLabs. KanMX-vox-CEN3-vox and hphNT1-vox-CEN3-vox were synthesized by Genewiz. (the distance between the left vox and CENs is 100 bp; the distance between the right vox and CENs is 123 bp). The 1 kb homologous segments near the

centromeres of the wild-type chromosome were PCR amplified from the genome of BY4741. wtII-hphNT1-vox-CEN3-vox, wtV-hphNT1-vox-CEN3-vox, wtVI-hphNT1-vox-CEN3-vox, wtIII-KanMX-vox-CEN3-vox, wtIX-KanMX-vox-CEN3-vox, and wtX-KanMX-vox-CEN3-vox were assembled into pUC19 linearized by *Sal*I and *Bam*HI digestion via Gibson assembly [7]. These sequences are provided in Table S6.

Yeast transformation

Yeast transformations in this study were performed with LiAc/SS/PEG method [8]. Briefly, the overnight YPD saturate culture of yeast cells were diluted into 5 mL fresh YPD medium for another 4-6 h culture starting from A_{600} of 0.2 at 30 ℃ in a shaker incubator at 220 rpm. Cells were washed with ddH_2O followed by 0.1 M LiAc, and chilled on ice. 620 μL of 50 % polyethylene glycol (PEG-3350), 40 μL of salmon sperm DNA (100 mg/mL), 90 μL of 1 M LiAc, and 150 μL plasmid solution (100 ng DNA) were added in order to the cell pellet. The mixture was vortexed briefly to resuspend the cells and incubated at 30 °C for 30 min. 90 μL of dimethyl sulfoxide (DMSO) was then added to the mixture and a heat-shock for 18 min at 42 ℃ was performed. Cells were then collected and resuspended with 5 mM $CaCl_2$. 100 μL of the cell suspension was then plated on SC–His for the selection of transformants. For drug selection marker, the cells were washed once with YPD and resuspended in 500 μL of YPD. They were then incubated on a shaker for 4-6 h at 30 ℃ and spread on a plate with the appropriate dominant drug selection marker. YPD, yeast extract

peptone dextrose; SC, synthetic complete medium. Geneticin Selective Reagent (G418, 200 mg/L) was added to YPD to select for kanMX4. Then, 200 mg/L hygromycin B was added to SC to select for hphNT1.

Yeast mating process

Yeast strains of opposite mating types were incubated in selective medium overnight. Five hundred microliters of each culture were washed with ddH_2O, and the pellets were resuspended in 3 mL of YPD, and incubated at 30 ℃ in a shaker incubator at 220 rpm for 8 h. One hundred microliters of the mating suspension were subsequently spread on a selective medium agar plate for single colonies after incubation at 30 ℃ for 2 days. PCR screening of *MAT*a and *MATα* was performed to select *MAT*a/α diploid colonies. Forward primer of *MAT*a “ACTCCACTTCAAGTAAGAGTTTG”: forward primer of *MAT*α “GCACGGAATATGGGACTACTTCG”; reverse primer of mating type “AGTCACATCAAGATCGTTTATGG”.

PCRTag assay for native chromosome elimination

PCRTags are landmarks in synthetic chromosomes including the peritelomeric and pericentromeric regions [9]. The loss of wild-type PCRTags associated with native chromosomes and the retained synthetic PCRTags in synthetic chromosomes were validated by colony PCR to assess the elimination of wtII, III, V, VI, IX, and X, using the primers listed in Supplementary Table 5.

Construction of a poly-synthetic strain using chromosome elimination with Vika/vox

First, pUC19-wtII-hphNT1-Vox and pUC19-wtIX-KanMX-vox-CEN3-vox were linearized by *Sal*I and *Bam*HI to obtain the wtII-hphNT1-Vox and wtIX-KanMX-vox-CEN3-vox constructs. Then, the prepared constructs were directly used for two yeast transformations in strain yYW169 (synV, synX) followed by successive selection on SC+hygromycin B medium and YPD+G418 successively. This strain was then mated with strain yZY192 (synII, synIII, synVI and synIXR). Heterozygous diploid strains were transformed with a pGAL-Vika plasmid with the *LEU2* auxotrophic marker [10], and then inoculated into liquid YP+galactose (2%) medium for 12-18 h at 30 ℃ to turn on the Vika/vox recombination system. Native chromosome II and IX were completely eliminated after Vika recombinase expression was induced. Culture samples of 200 μL were spread on plates with SC–Leu with dextrose medium. After incubation for 2 days at 30 ℃, single colonies were replicated on the plates containing YPD+G418 and SC+hygromycin B medium, to identify the colonies that had lost native chromosomes II and IX. The PCRtag assays described above were used to assess the elimination of native chromosomes. Following the same strategy, wtV, wtX, wtVI, and wtIII were eliminated sequentially, generating a 2n–6 strain. The yYW393 strain was cultured in YPD for 24 h to allow endoreduplication.

Meiosis and sporulation

The yYW393 strain was first transformed with a plasmid containing the *MAT**a*** and *HIS3* markers. Yeasts were incubated in SC–His medium overnight at 30 ℃ with shaking at 220 rpm, after which 200 μL of the culture was transferred to 3 mL of YPD and then incubated at 30 ℃ with shaking at 220 rpm for 14 h for early stationary phase culture. The cells were washed with sterile ddH_2O three times. One milliliter of 50× sporulation medium, 500 μL of the required amino acids (2 g/L uracil, 10 g/L leucine), and 150 μL of 10% yeast extract were mixed together and then diluted with sterile ddH_2O to a volume of 50 mL. All washed cells were transferred into sporulation solution, mixed well, and subjected to sporulation at room temperature for 5 days followed by dissection. To digest asci, 50 µL cell cultures samples were pelleted by centrifugation (2,000 g × 1 min), re-suspended in 50 µl 0.5 mg/mL 20T zymolyase in 1 M sorbitol at 37 °C for 10 min, and diluted with 300 µl 1 M sorbitol. Tetrads were dissected under a SINGER Instruments dissection microscope and germinated on YPD plates at 30 ℃ for 2 days. Nearly 100 tetrads were dismantled to produce one viable haploid. Yeast circular chromosome can cause dicentric chromosome and low spore viability [11, 12]. Thus synVI circulation could have been the main cause of observed defective germination. Removal of partial tRNAs may be another reason that affects germination. We also detected 35 mutations including SNPs and Indels in the yYW394 strain. Some of these mutations may have acted as suppressors of defective germination, which is under further investigation in our laboratory.

Adaptive laboratory evolution of yYW394

The yYW394 strain was inoculated in a 5 mL YPD liquid medium with a starting OD600 of 0.1 and grown in a 33 °C shaken incubator at 220 rpm for 24 hours. The cell suspension was then diluted 100-fold into a fresh 5 mL YPD medium, and cultured under the same conditions except that the temperature was set 1 °C higher (34 °C). This process was repeated by increasing the culturing temperature 1 °C at a time until 39 °C. After 24 h culture at 39 °C, the cell suspension was diluted 10,000-fold in ddH2O and 200 μL was evenly spread on a YPD agar plate, which was then placed in a 39 °C incubator for 3 days. From ~30,000 colonies on 15 plates, 56 large colonies were selected and subjected for plate spot assays at 30 °C and 37 °C with wild type BY4742 as a control. One out of 56 colonies exhibiting the most robust growth similar to the wild type level at both temperatures was named yZSJ025. Genome sequencing of yZSJ025 in reference to yYW394 identified 38 mutations on synthetic chromosomes and 16 on native chromosomes. Among the 54 mutations, 46 occurred in the ORFs of 24 genes and the rest 8 mutations are located in non-coding regions. All mutations were summarized in Tables S2 and S3.

Preparation of the SCRaMbLEd pool

The SCRaMbLE experiment using pCLB2-Cre-EBD-CYC1t was performed as described previously [13]. Briefly, the yeast strain was transformed with the pCLB2-Cre-EBD-CYC1t plasmid, and selected on SC–His agar plates. A single colony was grown in SC–His medium at 30 °C for 16-20 h to serve as the inoculum.

Saturated cultures were diluted to an OD600 of 0.5 and inoculated into 50 ml fresh SC–His medium containing 1 μM β-estradiol (Sigma-Aldrich). The cultures were incubated at 30 ℃ for 8 h to turn on Cre activity in the cells and begin SCRaMbLE progression. Yeast cells were then harvested by centrifugation at 2,000 ×g, washed twice with ddH_2O to remove β-estradiol, resuspended in 50 mL of YPD liquid medium, and incubated for 24 h to generate the pool of SCRaMbLEd cells for further analysis. The pool of SCRaMbLEd cells was used for high-depth whole-genome sequencing after DNA extraction

High-depth whole-genome sequencing

After DNA extraction, 1 μg of genomic DNA was randomly fragmented by using glass bead, followed by fragment selection with the MGIEasyTM Universal DNA Library Prep Kit V1.0 (MGI, Cat number: 1000006985) to obtain an average size of 200-400 bp. The selected fragments were end repaired and 3'adenylated, and the adaptors were then ligated to the ends of these 3'adenylated fragments. The products were purified with the Agencourt AMPure XP-Medium kit. The purified double stranded DNAs were heat denatured to single stranded, and then circularized using the splint oligo sequences. The sequencing libraries were constructed without PCR amplification. Single stranded circular DNAs (ssCir DNAs) were sequenced by BGISEQ-500. Finally, pair-end 150 bp reads were obtained by combinatorial Probe-Anchor Synthesis (cPAS). Furthermore, to accurately estimate the copy number of target regions, amplification-free sequencing was applied to decrease the

likelihood that an appreciable proportion of these sequences would be duplicated and preserve a more even distribution of read coverage across the targeted sequencing regions.

Analyses of novel junctions

Structural variations in the synthetic chromosomes were identified by the alignment of loxPsym sites and neighboring sequences to the yZSJ025 reference genome sequence [14]. Reads containing loxPsym sites and the adjacent sequences of 116 bp on both sides were extracted. The following criteria were used to identify and screen rearrangements for further studies: (1) reads containing the entire 34 bp loxPsym site sequences with flanking sequences belonging to two loxPsym sites of the reference; (2) reads with one end located less than 4 bp from a loxPsym were excluded; and (3) reads containing two or more mismatched bases were excluded.

Identical reads were considered as a result of a single rearrangement event. Only two or more reads supporting a rearrangement event were included in the further analyses. Matlab package associated with analyses of novel junctions is available at https://github.com/Limin-Jiang/Analyses-of-novel-junctions.

RNA-Seq Analysis

Yeast strain yZSJ025 was grown from single colonies in liquid YPD culture until reaching early-log phase (OD_{600} = 1) at 30 ℃ with rotation. Total RNA was extracted using the Trizol (Invitrogen, CA, USA). Three parallel samples were set for each

yeast strain. The samples were tested using the Illumina HiSeq 4000 platform. HISAT2 [15] (V2.1.0) was used to compare clean reads with the reference genome [15], and Bowtie2 (V2.3.5) was used to align the quality-controlled sequence to the reference transcription sequence. Using RSEM [16], the bowtie2 comparison results were used for statistics, and the number of reads compared to each transcript of each sample was obtained, and FPKM [17] (Fragments Per Kilobase Per Million bases) conversion was performed.

ATAC-seq

Yeast strain yZSJ025 was grown from single colonies in liquid YPD culture until reaching the stationary phase at 30 ℃ with rotation. A total of ~1,000,000 yeast cells were washed with 1 mL RSB buffer (10 mM Tris-HCl pH 7.4, 10 mM NaCl, 3 mM $MgCl_2$) once and incubated for 60 min at 37 ℃ using zymolyase to digest cell walls (0.015 g/mL, Solarbio). Then the cells were resuspended in 1 mL lysis buffer (10 mM Tris-HCl pH 7.4, 10 mM NaCl, 3 mM $MgCl_2$, 0.5% NP40, 0.1% digitonin, 0.1% Tween-20, 1x protease inhibitor) for 10 min at 4 ℃ to lyse the yeast cell membrane to obtain the nucleus. Immediately following nuclear preparation, the pellet was resuspended in the Tn5 transposase reaction mix. The transposition reaction was carried out for 30 min at 37 ℃. Tn5 transposed DNA was purified by AMPure DNA magnetic beads. A qPCR reaction was performed on a subset of the DNA to determine the optimum number of PCR cycles. The amplified libraries were evaluated on an Agilent Tapestation 2100 (Agilent Technologies) to detect size distribution of

library fragments. Biological replicates were performed in duplicate for all ATAC experiments. The final library was sequenced on an Illumina Nova-seq PE150 platform. Peaks in replicates and self-pseudoreplicates were called using MACS2 [18] (--nomodel --extsize 200 --shift -100). Nucleoatac version 0.3.4 was used to call nucleosome positions and occupancy by ATAC data with the default parameters.

Analyses of the ATAC-seq signals

Prior to mapping, standard next-generation sequencing quality control steps using Trimmomatic(version 0.38) with parallels ILLUMINACLIP: Trimmomatic/adapters/NexteraPE-PE.fa:2:30:10:8:true LEADING:3 TRAILING:3 SLIDINGWINDOW:4:15 MINLEN:8 were performed. Trimmed reads were aligned to reference genomes using Bowtie2(version 2.2.6) with parallels -X 2000 --dovetail. PCR duplicates in the samples were removed using Picard(version 1.126) (http://broadinstitute.github.io/picard/). Peaks were called, and graphs of genomic accessibility were generated using MACS2 (3) (--nomodel --extsize 150 --shift -75). Nucleosome occupancy was generated using Nucleoatac(v0.3.4) [19]. The average ATAC-seq signals of loxPsym site were generated using deepTools (v 3.5.1). The mean ATAC-seq signals in the 400 bp surrounding hotspots and coldspots were normalized according to the mean signal of all 877 loxPsym sites as follows：

$$\text{Normalized signal}=\frac{\text{Mean hotspot (or coldspot) signal}}{\text{Mean signal of all 877 loxPsym sites}}$$

Hi-C sequencing

Yeast strain yZSJ025 was grown from single colonies in liquid YPD culture until reaching the stationary phase at 30 ℃ with rotation. The genomic DNA from exponential phase cells was cross-linked and digested with 200U *Mbo*I (NEB) as previously described [20]. Restriction fragment ends were labeled with biotinylated cytosine nucleotides by biotin-14-dCTP (TriLINK) followed by ligation. Purified DNA was sheared to a length of ~400 bp. Point ligation junctions were pulled down with Dynabeads MyOne Streptavidin C1 (Thermo Fisher). The Hi-C library for Illumina sequencing was prepared by using the NEBNext Ultra II DNA library Prep Kit for Illumina (NEB Cat# E7645S) according to the manufacturers' instructions. Fragments between 400 and 600 bp were paired-end sequenced on the Illumina Nova-seq PE150 platform.

Construction of the contact map and chromosome 3D model

After quality filtering using Trimmomatic (version 0.38), the clean Hi-C data of two biological replicates for sample yZSJ025, were iteratively mapped to the yZSJ025 genome using the ICE software package (version 1f8815d0cc9e). Dangling ends and other unusable data were filtered, the valid pairs were used to analyze the correlation efficiency of the two biological replicates for each sample using QuASAR-Rep analysis (3DChromatin-ReplicateQC v0.0.1). Then we pooled the data from two replicates together for further analysis. Valid pairs after pooling were binned into 1 kb and 10 kb nonoverlapping genomic intervals to generate contact maps. Raw Hi-C contact maps were normalized using the iterative normalization method to eliminate

systematic biases. The chromosomal 3D structure of the strain was inferred using the Pastis (v0.1) method [21]. with a multidimensional scaling (MDS) model. The 10-kb contact maps were used to construct the 3D model.

Statistical analysis

Statistical analysis was performed using R (http://www.r-project.org/) or GraphPad Prism 8.0. Significance was determined via two-tailed, two-sample t tests. Pearson correlation analysis was applied to determine the correlation coefficient (r) and associated *p* values. A p value of ≤ 0.05 was considered statistically significant.

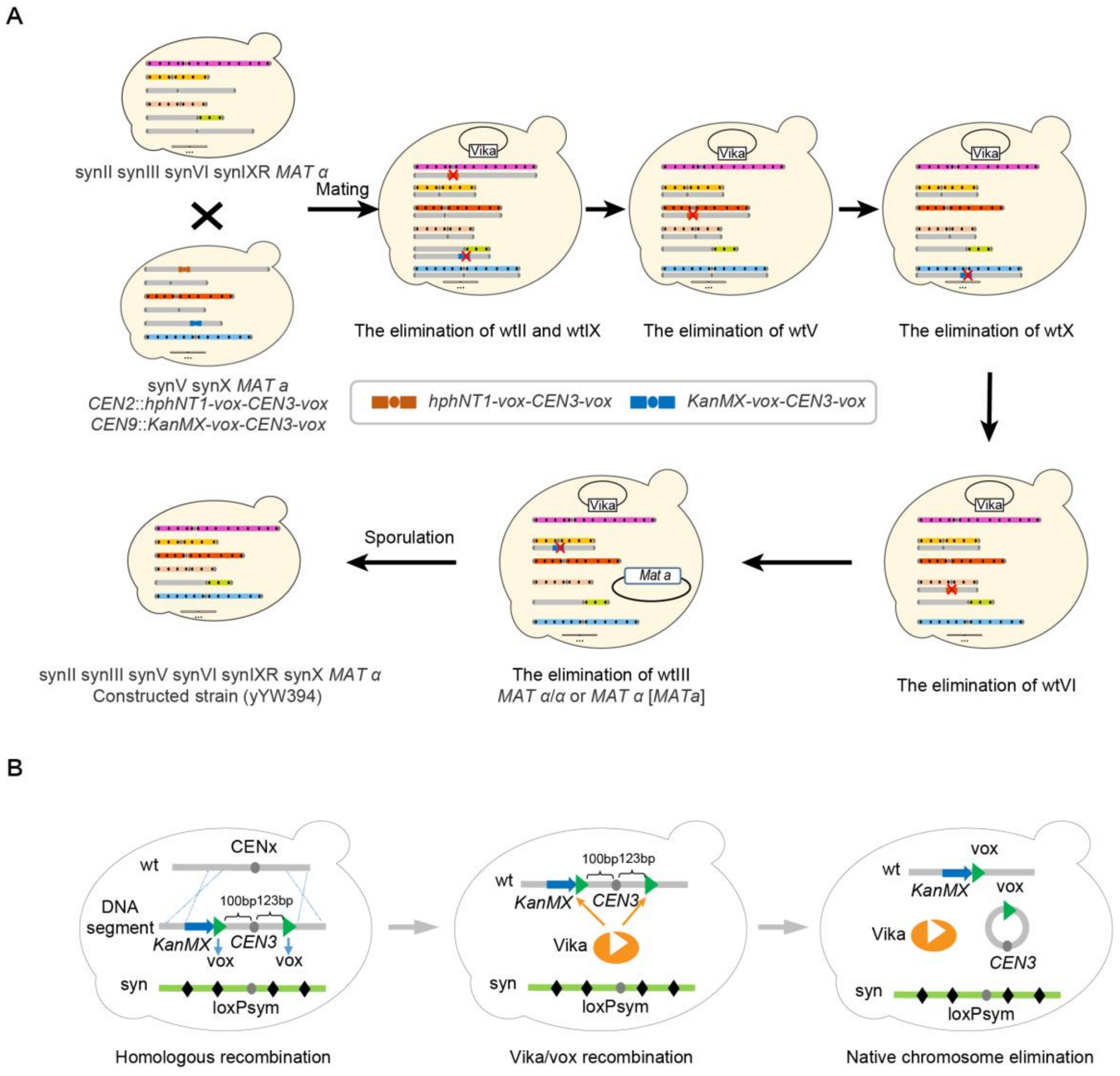

Figure S1. Consolidation of a poly-synthetic chromosome strain. **(A)** *KanMX-vox-CEN3-vox* and *hphNT1-vox-CEN3-vox* constructs were integrated into the centromere regions of native chromosomes II and IX in strain yYW169 (synV, synX), and the resulting strains were then mated with strain yZY192 (synII, synIII, ring_synVI and synIXR). When Vika recombinase was induced, the native chromosomes were eliminated and confirmed by PCRTag analysis. Following the same strategy, wtV, wtX, wtVI, and wtIII were eliminated sequentially. An episomal copy of *MATa* was introduced to permit sporulation after elimination. A haploid

synthetic chromosome strain (yYW394) was then generated by sporulation. Synthetic chromosomes are labeled with different colors and the native chromosomes are shown in gray. **(B)** Mechanism for chromosome elimination with Vika/vox. The centromere constructs contain two vox sites, *CEN3* and *KanMX* or *hphNT1*. The constructs were integrated into the centromere regions of native chromosomes. After growth on galactose to induce the destabilization of native chromosomes，the strains were spread on plates with SC–Leu with dextrose medium. The 2n–2 state was cultured in YPD for 24 h allowing endoreduplication and then confirmed by PCRTag analysis. SynVI is a ring chromosome.

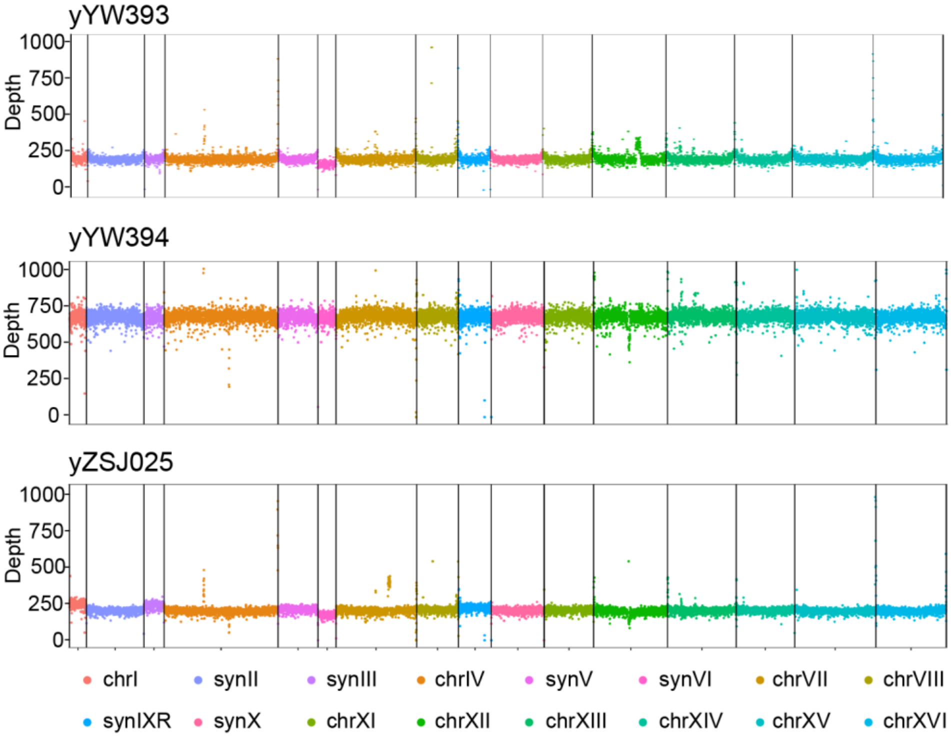

Figure S2. The coverage maps of yYW393, yYW394 and yZSJ025. The sequencing depths of all sixteen chromosomes are shown at the same scale. No major structural variations were detected in either strain. The SNPs and short indels were listed in Table S2 and Table S3. SynVI was tentatively identified as a ring chromosome. Ring chromosomes tend to have a lower sequencing coverage in NGS analysis, which is observable for synVI in yYW393 and yZSJ025.

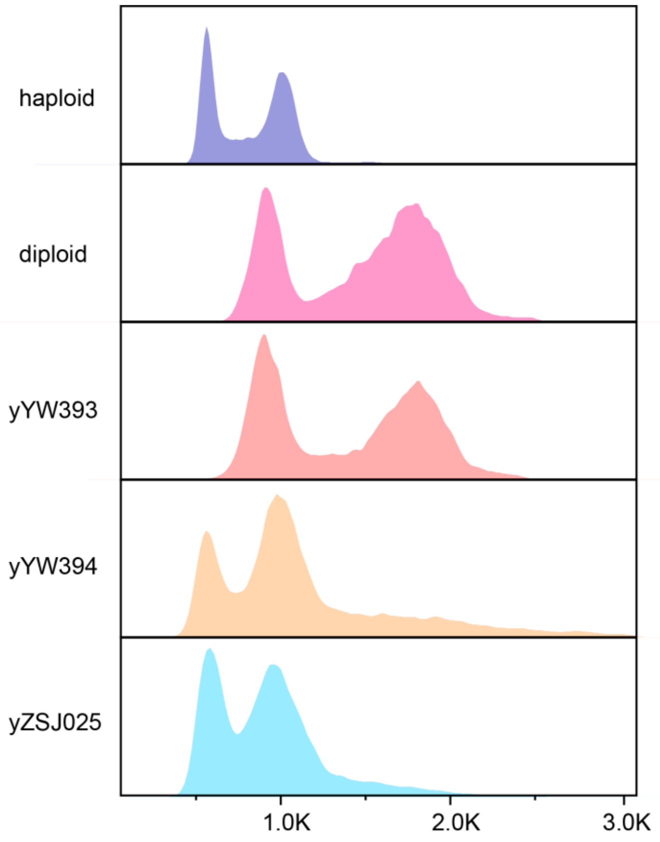

Figure S3. Cell ploidy of yYW393, yYW394 and yZSJ025 identified via flow cytometry. Although both yYW394 and yZSJ025 are confirmed haploid, the pattern of yZSJ025 is more similar to that of the wild type haploid strain. yYW394 exhibits more severe G2/M arrest phenotypes and more cells in abnormally higher ploidy states. This is consistent with recovered growth fitness of yZSJ025.

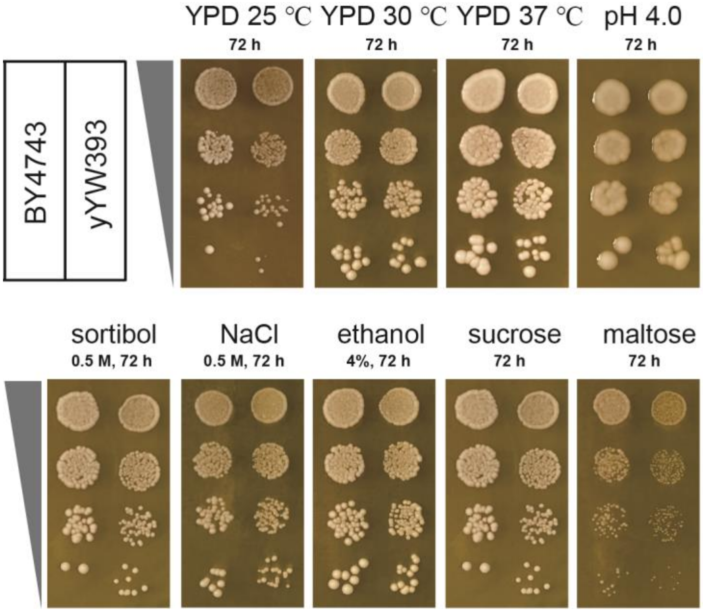

Figure S4. Spotting assays to characterize the growth phenotypes of yYW393. Cells of indicated genotypes were washed in water, and serial dilutions (1:10) of 1×10^7 cells were inoculated onto agar plates as indicated and allowed to grow for 72 hours before the plates were photographed.

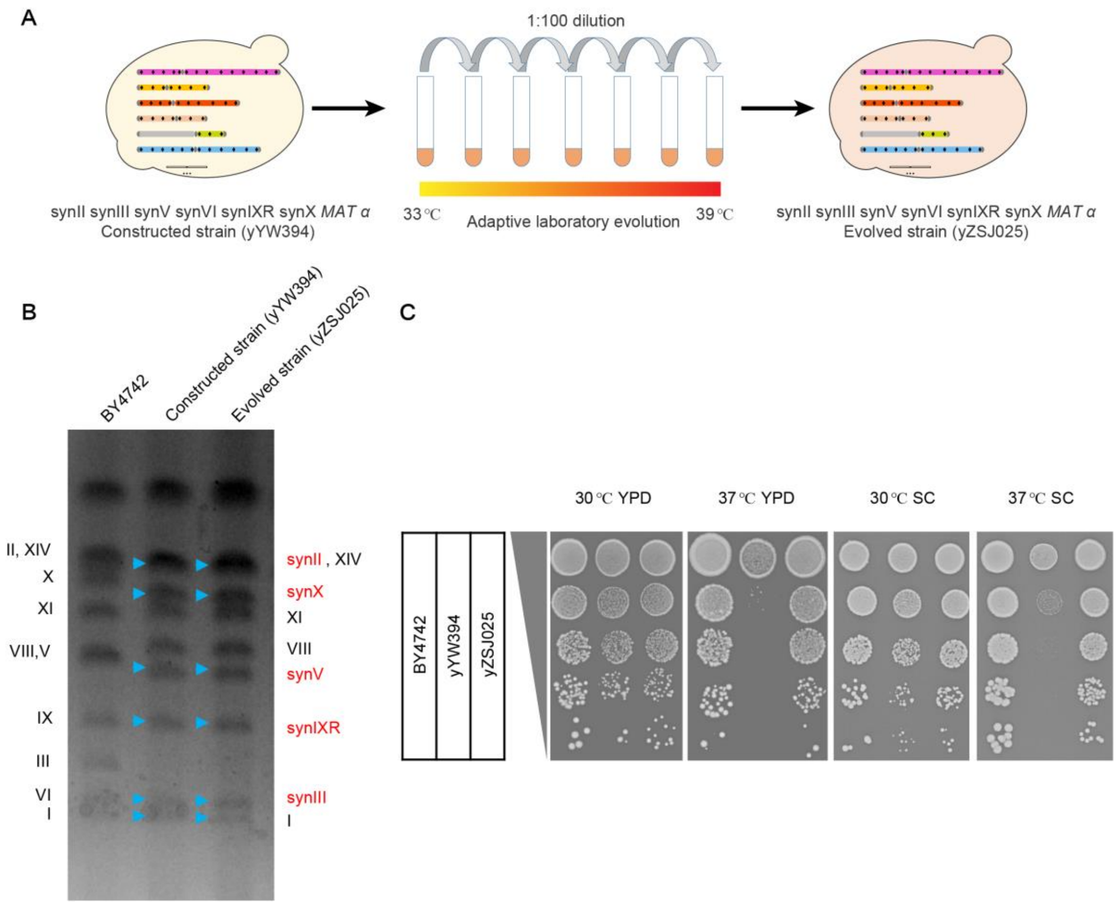

Figure S5. The phenotypes and genotypes of yYW394 and yZSJ025. **(A)** Adaptive laboratory evolution of yYW394 was carried out from 33 ℃ to 39 ℃ to generate an evolutionary haploid strain (yZSJ025). Synthetic chromosomes are labeled with different colors and the native chromosomes are shown in gray. **(B)** Karyotypic analysis of BY4742, yYW394 and yZSJ025 by pulsed-field gel electrophoresis; note that IXL-synIXR and native IX migrate identically. Consistent with a previous report [1] that circular DNA is not able to migrate into the gel and therefore retains in the well, circular synVI is also not visible in our gel. **(C)** Spotting assays to characterize the growth phenotypes of yYW394 and yZSJ025.Ten-fold serial dilutions of BY4742, yYW394 and yZSJ025 on various types of media at 30 ℃ or 37 ℃. YPD, yeast

extract peptone dextrose; SC, synthetic complete medium.

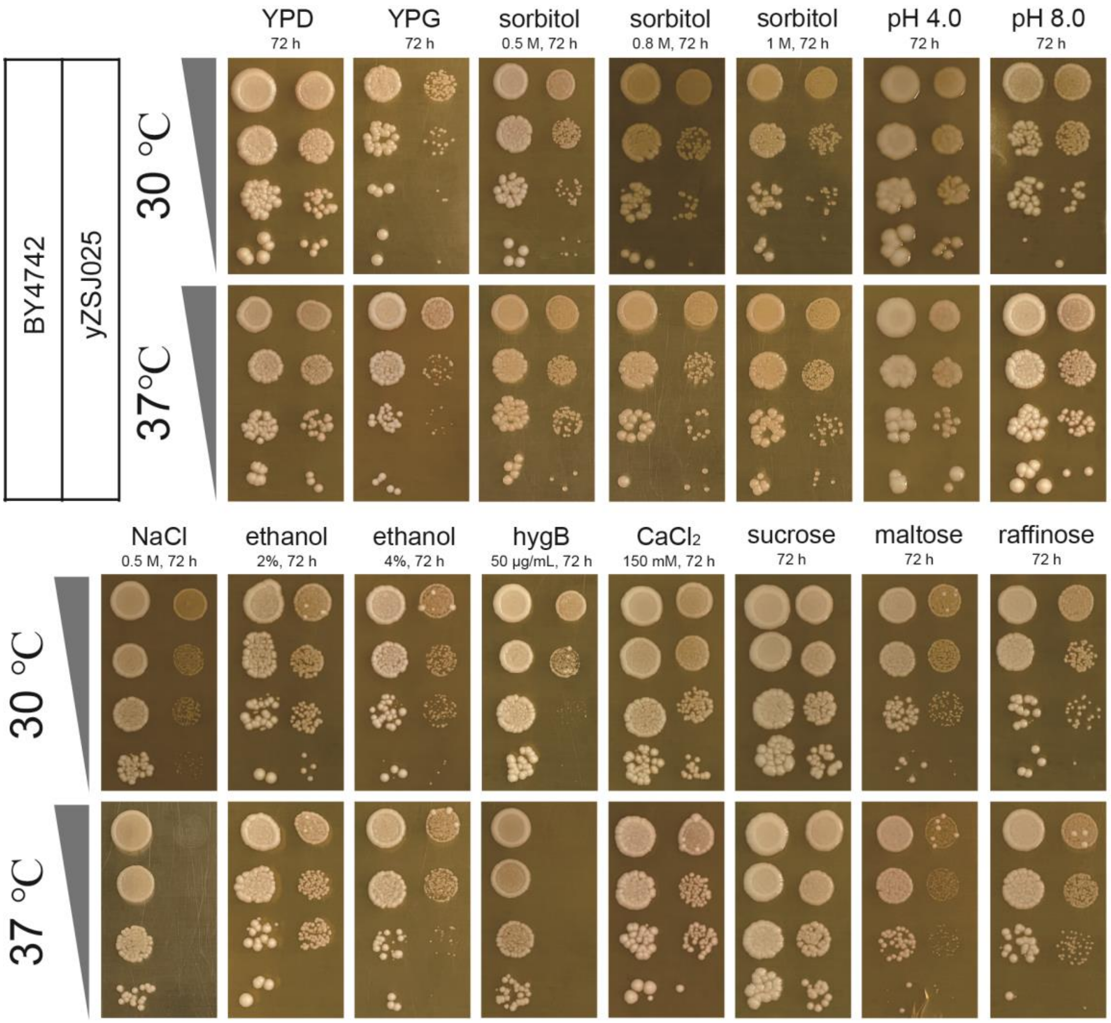

Figure S6. Spotting assays to characterize the growth phenotypes of yZSJ025. Cells of indicated genotypes were washed in water, and serial dilutions (1:10) of 1×10^7 cells were inoculated onto agar plates as indicated and allowed to grow for 72 hours before the plates were photographed.

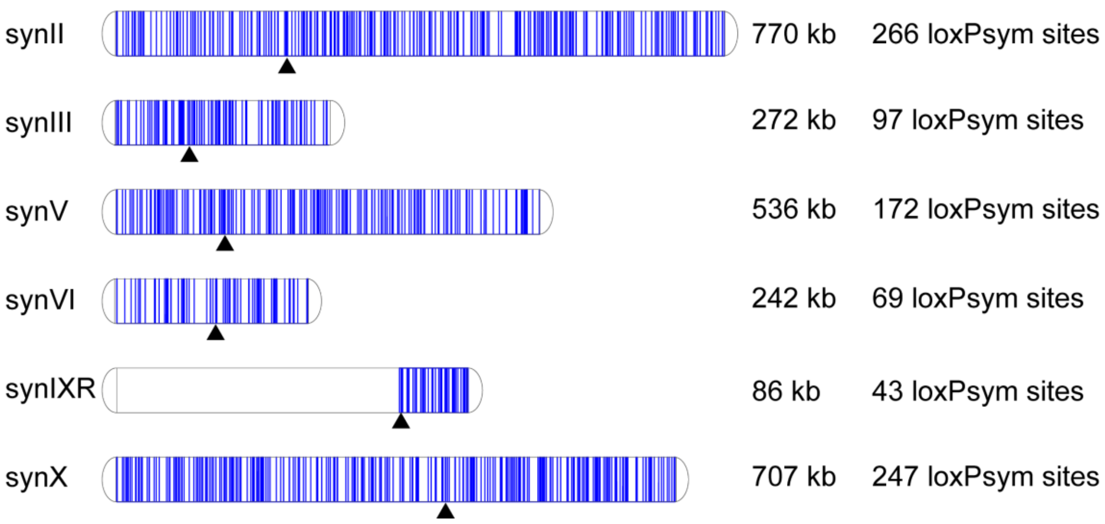

Figure S7. Distributions of 894 loxPsym sites. Seventeen loxPsym sites were inserted at the synthetic telomere regions, where flanking sequences were not distinguishable from each other; these sites were excluded from the identification of rearrangement reads. The blue lines indicate loxpsym sites, while triangles indicate the centromeres.

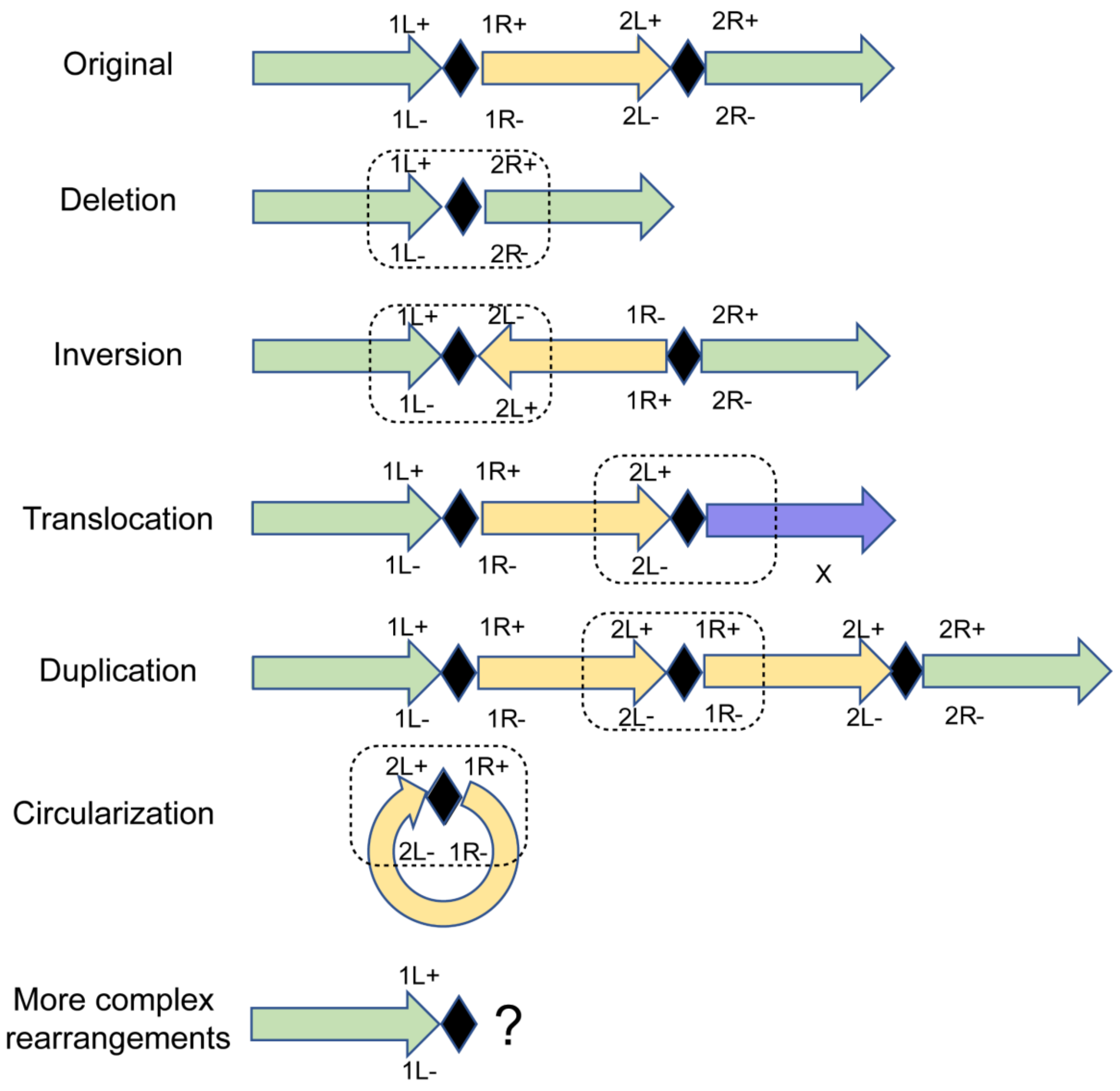

Figure S8. Classification strategy for novel junction analysis. “1” and “2” indicate the order of the segment in a synthetic chromosome; “X” indicates the segment in another synthetic chromosome; “+” and “-” indicate the positive and negative strands. Deletion events could be identified as a “1L+ & 2R+” or “1L- & 2R-” connection type, inversion events could be identified as a “1L+ & 2L-” or “1L- & 2L+” connection type, and translocation events could be identified as a “2L+ & X” or “2L- & X” connection type. Other types of junctions could be identified as evidence of complex events (e.g., duplication and circularization: “2L+ & 1R+” or “2L- & 1R-”).

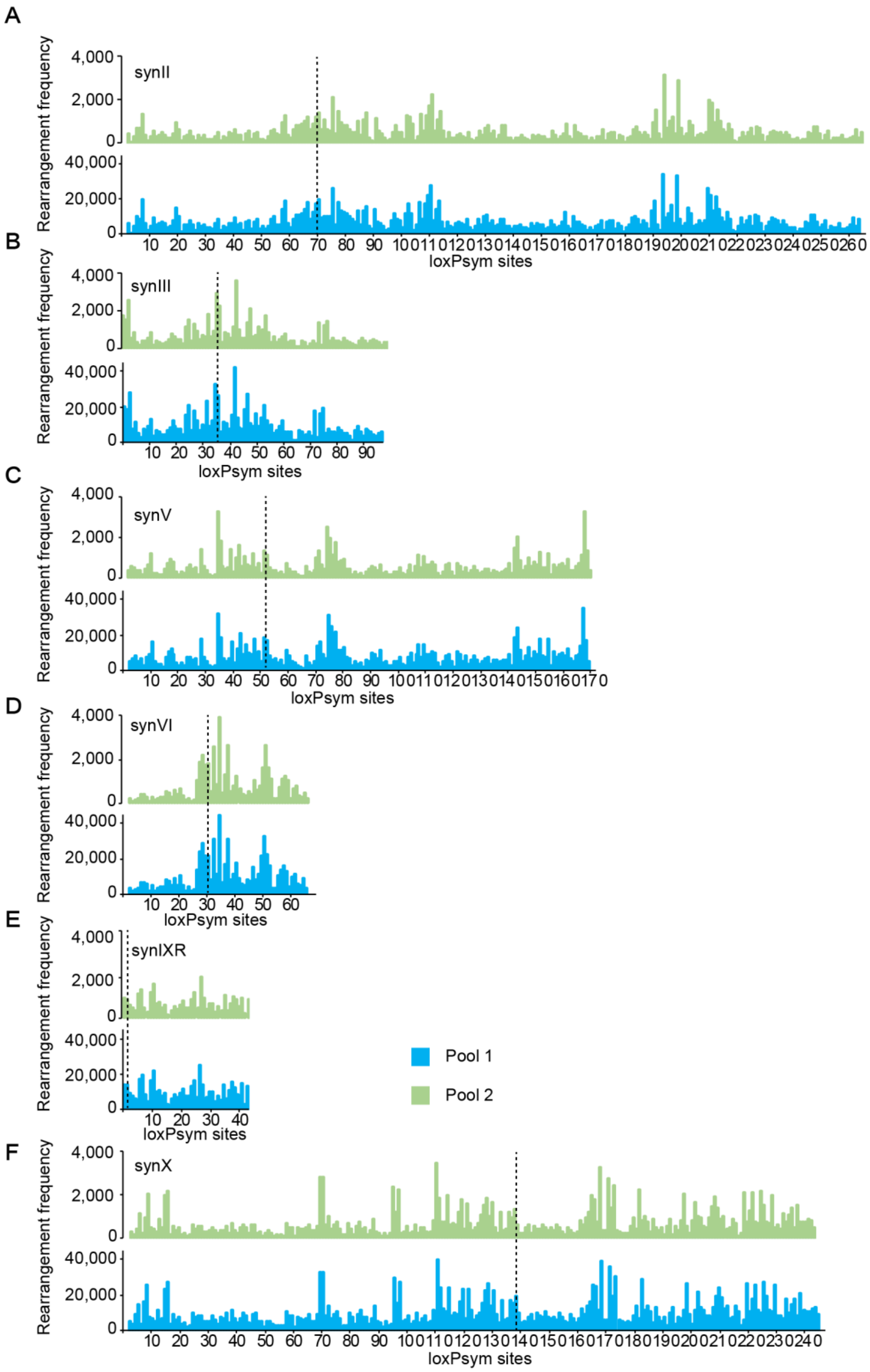

A
B
C
D
E
F
synII
synIII
synV
synVI
synIXR
synX
Rearrangement frequency
loxPsym sites
Pool 1
Pool 2

Figure S9. Landscape of the rearrangement frequencies along synII, synIII, synV, synVI, synIXR, and synX in biological replicates. The dotted line indicates centromeres. SynVI is a ring chromosome.

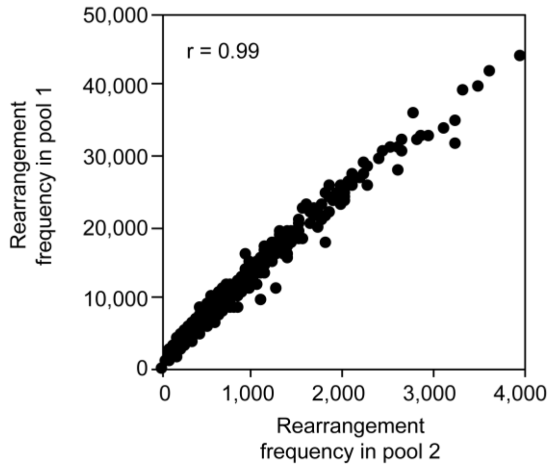

Figure S10. Comparison of rearrangement frequencies in the replicates of SCRaMbLEd cells (r = 0.99, p = 1.7×10^{-232}). Pearson correlation analysis was applied to determine the correlation coefficient and associated p values.

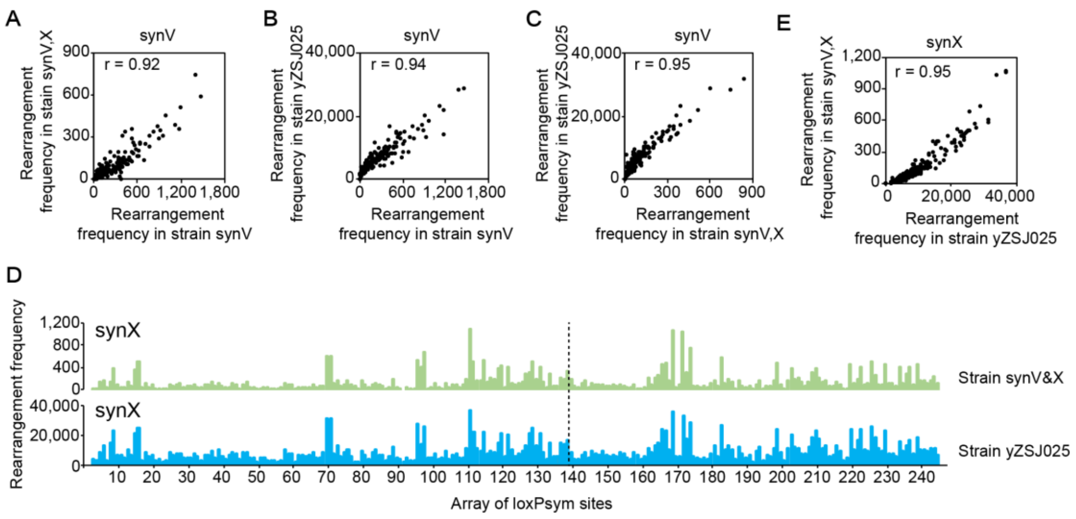

Figure S11. Intra-chromosomal rearrangement patterns of synV and synX. **(A-C)** Comparisons of rearrangement frequencies in synV following the SCRaMbLEing of strains yXZX846 (synV), yYW169 (synV, synX) and yZSJ025. **(A)** Comparisons of strains yXZX846 and yYW169 (r = 0.92, $p = 5.1\times10^{-12}$). **(B)** Comparisons of strains yXZX846 and yZSJ025 (r = 0.94, $p = 6.0\times10^{-49}$). **(C)** Comparisons of strains yYW169 and yZSJ025 (r = 0.95, $p = 3.9\times10^{-51}$). **(D)** Intra-chromosomal rearrangement patterns of synX following SCRaMbLEing in two different yeast strains, yYW169 and yZSJ025. The red dots indicate centromeres. **(E)** Comparisons of rearrangement frequencies in synX following the SCRaMbLEing of strains yYW169 and yZSJ025 (r = 0.95, $p = 1.6\times10^{-71}$). Pearson correlation analysis was applied to determine the correlation coefficient and associated p values.

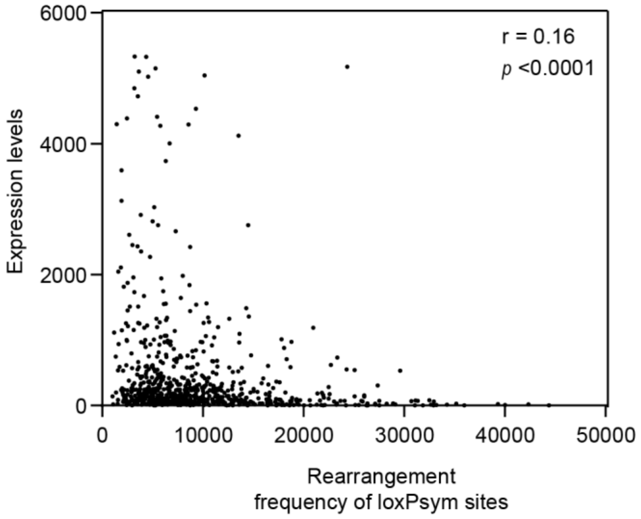

Figure S12. Comparisons of gene transcription and adjacent recombination frequency. (r = 0.16, $p < 0.0001$). Pearson correlation analysis was applied to determine the correlation coefficient and associated p values.

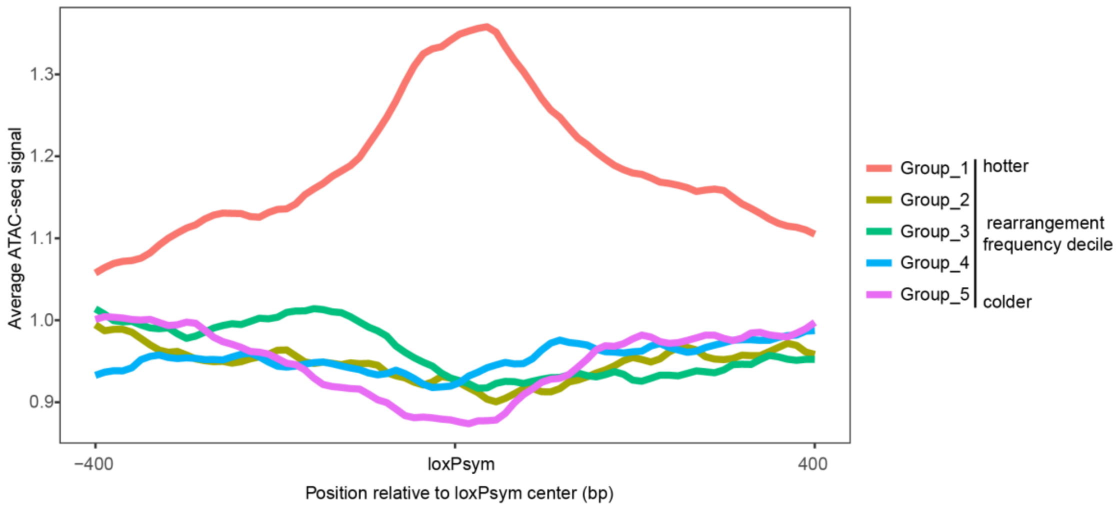

Figure S13. Average ATAC-seq signals of loxPsym-centered 800 bp regions. LoxPsym sites were divided into quintiles according to rearrangement frequency. Average ATAC-seq signals of each group can be compared among the five groups. ATAC signals correlate well to the rearrangement frequencies, with the highest ATAC signals observed for Group 1, the lowest for Group 5 and intermediates for Group 2-4.

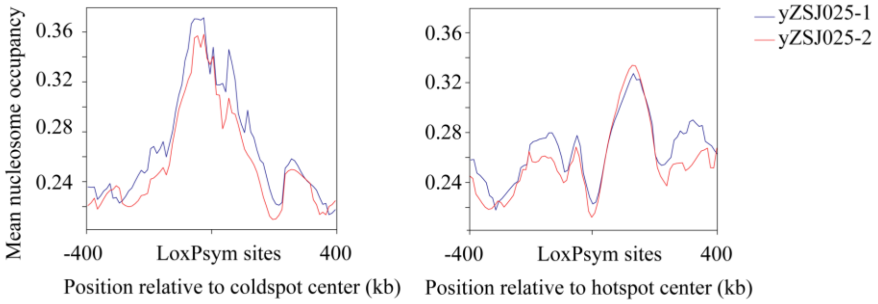

Figure S14. Mean nucleosome occupancy around hotspots and coldspots in two biological replicates, in blue and red respectively. The nucleosome occupancy was relatively low in hotspots and high in coldspots.

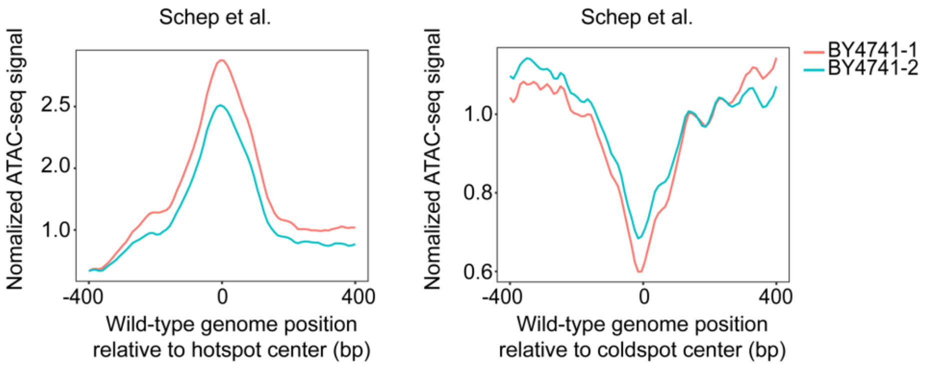

Figure S15. Normalized mean ATAC-seq signals around the wide-type chromosomal positions matching the hotspots and coldspots of synthetic chromosomes. Data were extracted from reported experiments performed with two different biological samples by the *Schep* research group [19]. ATAC-seq signals peaked at the positions of hotspots, and remained weak at coldspots on all six chromosomes of wild type strain (BY4741).

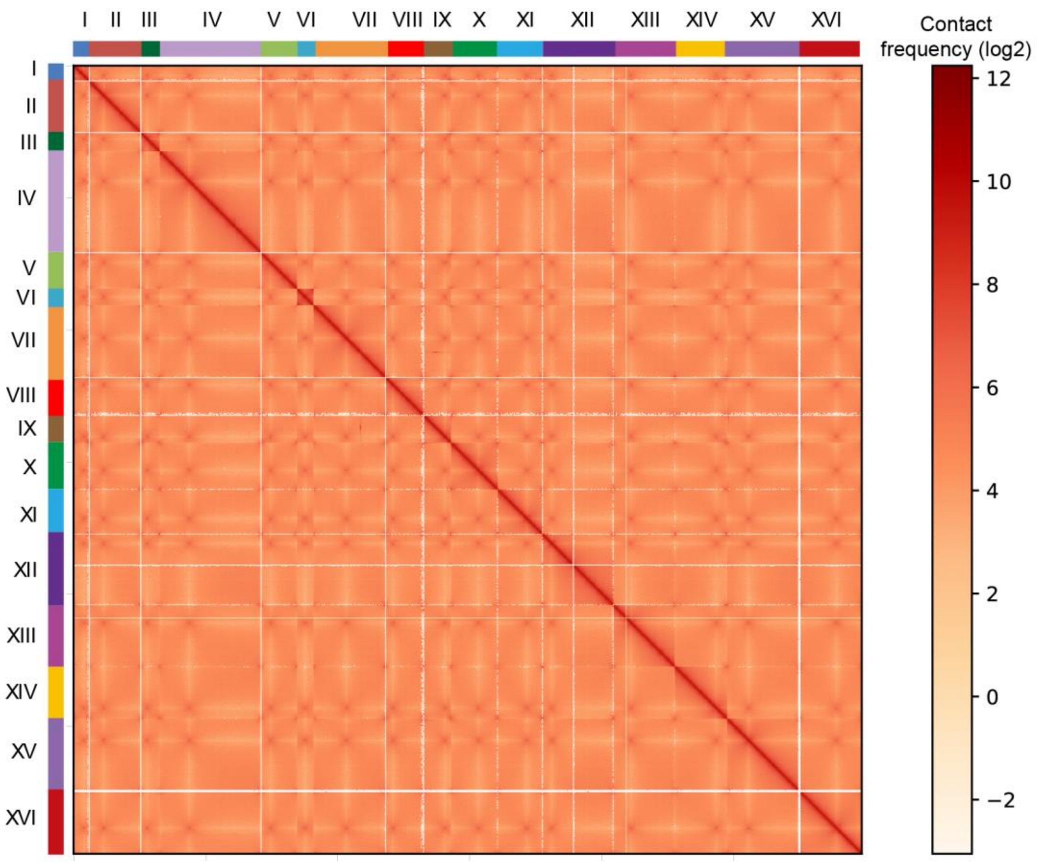

Figure S16. Contact map of yZSJ025. A normalized contact map of all chromosomes of yZSJ025 is shown (bin size, 10 kb). The normalized contact frequencies are indicated on a log2 scale with colors ranging from light yellow (few contacts) to red (many contacts). SynVI is a ring chromosome.

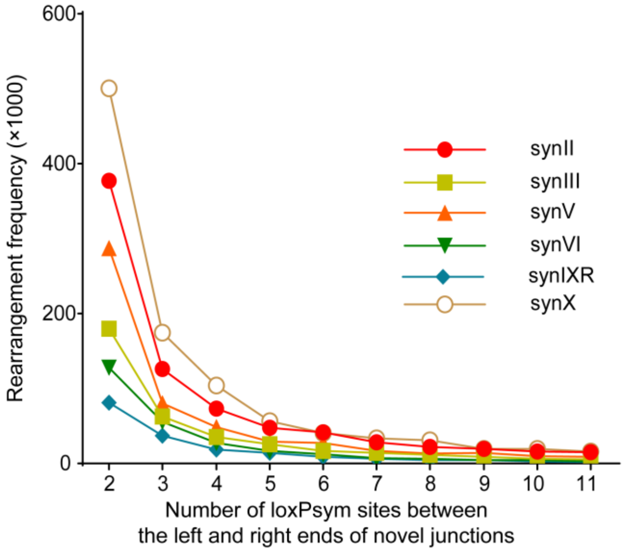

Figure S17. Distributions of the numbers of loxPsym sites between the left and right ends of novel junctions for all synthetic chromosomes. Rearrangements tended to occur most frequently between adjacent loxPsym sites on any of the synthetic chromosomes. Thus, the higher number of loxPsym sites is contained, the higher rearrangement frequency is observed for a synthetic chromosome.

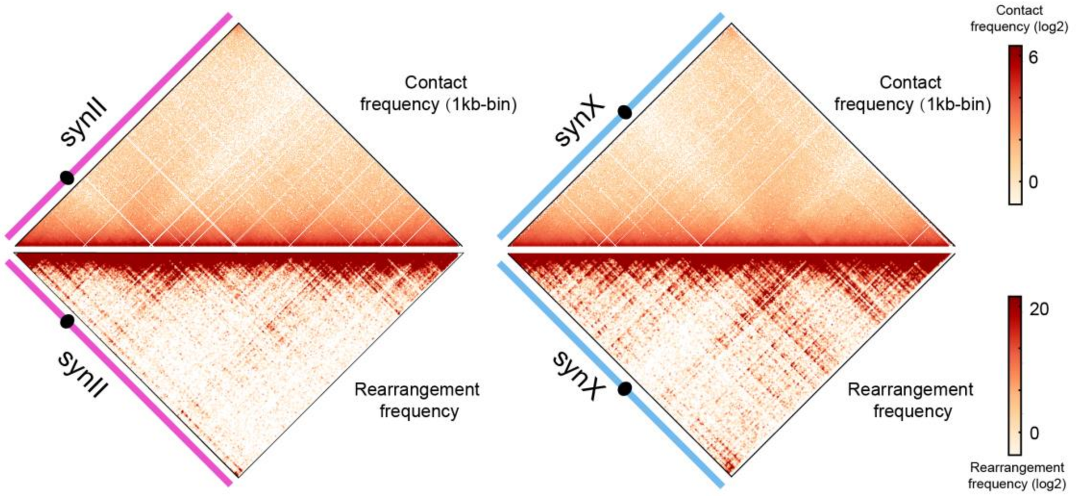

Figure S18. Hi-C heatmap and rearrangement frequency heatmap of synII and synX. Upper part: Hi-C heatmap. Spots with different heatmap values from low to high are colored from light yellow to red as indicated. Bottom part: rearrangement frequency heatmap. Spots with different heatmap values are labeled in red with different intensities as indicated.

Table S1. Summary statistics for synII, synIII, synV, synVI, synIXR, synX for strain yZSJ025.

Chromosome	SYN size (bp)	WT size (bp)	Number of loxPsym sites
chrII	770032	813184	266
chrIII	272194	316617	97
chrV	536024	576874	172
chrVI	242745	270148	69
chrIX	356000 (wtL)+86490 (synR)	439885 (wt IX)	43
chrX	707460	745751	247
Total	2614945	3162459	894

WT, wild type; SYN, synthetic.

Table S2. Sequence variants in yYW394.

Variation type	Chr	Position in ref_base	ref_base	yYW394	ORF	Amino acid Substitution
SNP	chr01	25340	C	A	YAL063C	Arg->Ser
SNP	chr01	25488	G	A	YAL063C	Synonymous
SNP	chr04	287316	G	T	YDL096C	Synonymous
SNP	chr04	1253397	T	C	YDR389W	Synonymous
SNP	chr04	783598	G	A	YDR164C	Synonymous
SNP	chr08	630505	A	G	YMR185W	Leu->Pro
SNP	chr14	520142	A	C	YNL054W-B	Synonymous
SNP	chr15	1273	G	A	YOL166C	Synonymous
SNP	chr15	1309	A	G	YOL166C	Synonymous
SNP	chr16	191944	C	T	YPL188W	Ser->Asn
SNP	syn02	126624	C	T	YBL040C	Pro->Leu
SNP	syn03	59244	T	C	Non-coding	Intergenic
SNP	syn03	67064	A	C	YCL025C	Asp->ALa
SNP	syn03	68242	G	T	YCL025C	ALa->Ser
SNP	syn03	177288	A	G	YCR040W	Synonymous
DEL	syn03	97056	CGC	C	Non-coding	Intergenic
INS	syn03	161496	A	ATTAAA AATTTG	YCR032W	Frameshift
INS	syn03	186156	G	GA	Non-coding	Intergenic
DEL	syn03	201317	TAT	T	Non-coding	Intergenic
DEL	syn03	221325	TC	T	YCR073C	Frameshift
SNP	syn05	3624	C	T	YEL072W	Synonymous
SNP	syn05	293628	G	A	YER075C	Asp->Ile
DEL	syn05	106824	ATA	A	Non-coding	Intergenic
DEL	syn05	268979	TAT	T	Non-coding	Intergenic
SNP	syn06	224818	T	C	YFR050C	.->Tyr
SNP	syn06	224926	A	G	YFR050C	Ala->Gly
SNP	syn06	224953	A	G	YFR050C	Ala->Gly
INS	syn06	43121	G	GTA	Non-coding	Intergenic
SNP	semi-syn09	417305	C	T	Non-coding	Intergenic
SNP	syn10	519025	G	A	YJR061W	Ala->Thr
SNP	syn10	568007	T	G	YJR092W	Synonymous
SNP	syn10	673441	G	A	YJR147W	Asp->Thr
INS	syn10	232012	T	TA	Non-coding	Intergenic
DEL	syn10	488591	GTG	G	Non-coding	Intergenic
DEL	syn10	607758	ACA	A	YJR115W	Frameshift
Circularity	syn06	The deletion of 649 bp of the left end of synVI and 689 bp of the right end of synVI				

Table S3. Sequence variants in yZSJ025.

Variation type	Chr	Position in ref_base	yYW394	yZSJ025	ORF	Amino acid Substitution
SNP	chr01	211021	T	C	Non-coding	Intergenic
SNP	chr04	1311961	A	G	Non-coding	Intergenic
SNP	chr04	1487318	C	G	Non-coding	Intergenic
SNP	chr07	362360	G	A	HNM1	Synonymous
SNP	chr07	401698	T	A	Non-coding	Intergenic
SNP	chr08	499956	T	C	PPX1	Synonymous
SNP	chr11	249055	G	A	HSL1	Val->Ile
SNP	chr11	414171	C	G	URB1	asp->his
SNP	chr12	732445	G	T	YLR302C	Gly->
SNP	chr12	941839	C	A	YLR410W-A/B	pro->
SNP	chr13	8157	G	A	COS3	Gly->Glu
SNP	chr14	605509	C	T	PBI2	Synonymous
SNP	chr14	628846	T	A	Non-coding	Intergenic
SNP	chr15	773074	C	G	MKK1	Asn->
SNP	chr16	186252	C	T	NAB3	Glu->Lys
SNP	chr16	324738	G	A	DBP1	Synonymous
SNP	syn02	91222	C	T	YBL061C	Glu->Phe
SNP	syn02	211489	G	A	YBL004W	Asp->Asn
INS	syn02	152448	T	TA	Non-coding	Intergenic
INS	syn03	67049	A	ATT	YCL025C	Frameshift
DEL	syn03	181993	CT	C	Non-coding	Intergenic
INS	syn03	186141	C	CA	Non-coding	Intergenic
SNP	syn05	229558	A	G	YER047C	Ser->Pro
SNP	syn05	229570	T	A	YER047C	Ser->Cys
SNP	syn05	468674	C	T	YER162C	GCG->Thr
INS	syn05	229553	T	TGC	YER047C	Frameshift
DEL	syn05	229560	CTT	C	YER047C	Frameshift
INS	syn05	229564	A	AGG	YER047C	Frameshift
DEL	syn05	229565	AC	A	YER047C	Frameshift
DEL	syn05	229568	GC	G	YER047C	Frameshift
DEL	syn05	504041	TTAG	T	YER173W	Frameshift
INS	syn05	504045	C	CTCA	YER173W	Frameshift
SNP	syn06	224938	A	G	YFR050C	Synonymous
SNP	syn06	224930	A	AGTG	YFR050C	Frameshift
DEL	syn06	224932	GCTA	G	YFR050C	
DEL	syn06	224942	CGGCTGCT	C	YFR050C	Frameshift
INS	syn06	224950	T	TAGAAG	YFR050C	Frameshift

				AA		
SNP	syn10	138855	G	A	YJL143W	Ala->Thr
SNP	syn10	377689	G	A	YJL025W	Met->Ile
SNP	syn10	410493	T	A	YJL005W	Leu->Gln
SNP	syn10	568017	G	A	YJR092W	Ala->Thr
SNP	syn10	568034	C	T	YJR092W	Synonymous
DEL	syn10	568009	TCAC	T	YJR092W	Frameshift
DEL	syn10	568019	TA	T	YJR092W	Frameshift
INS	syn10	568022	C	CATCT	YJR092W	Frameshift
INS	syn10	568030	A	ATT	YJR092W	Frameshift
DEL	syn10	568031	CAG	C	YJR092W	Frameshift
INS	syn10	572010	T	TA	YJR094C	Frameshift
INS	syn10	572011	G	GAA	YJR094C	Frameshift
DEL	syn10	572012	CT	C	YJR094C	Frameshift
DEL	syn10	572026	AT	A	YJR094C	Frameshift
INS	syn10	572029	G	GCC	YJR094C	Frameshift
DEL	syn10	572032	AGCG	A	YJR094C	Ala->
Circularity	syn06	The deletion of 649 bp of the left end of synVI and 689 bp of the right end of synVI				

Table S4. The plasmid and yeast strains used in this study.

Strain name	Description	Genotype
yXZX846	Haploid strain synV	*MAT a his3Δ1 leu2Δ0 ura3Δ0 met15Δ0 synV*
yYW169	Haploid strain synV&X	*MAT a his3Δ1 leu2Δ0 ura3Δ0 met15Δ0 SYNX HO::tR(CCU)J synV and X*
yYW0394	Haploid strain synII, III, V, ring_VI, IXR, X	*MAT alpha his3Δ1 leu2Δ0 ura3Δ0 IXL-synIXR SYNX HO::tR(CCU)J synII, III, V, ring_VI, IXR, and X*
yZSJ025	Directed evolution strain from yYW0394	*MAT alpha his3Δ1 leu2Δ0 ura3Δ0 IXL-synIXR SYNX HO::tR(CCU)J synII, III, V, ring_VI, IXR, and X*
yZY192	Haploid strain synII, III, ring_VI, IXR	*MAT alpha his3Δ1 leu2Δ0 ura3Δ0 IXL-synIXR synII, III, ring_VI, and IXR*
yYW268	yZY192&yYW169	*MAT alpha/a his3Δ1 leu2Δ0 ura3Δ0 IXL-synIXR SYNX HO::tR(CCU)J synII, III, V, ring_VI, IXR, and X*
bYW0185	pUC19-wtII-hphNT1-Vox	*Both sides containing 1kb homologous fragments of wild-type chromosome II*
bYW0186	pUC19-wtV-hphNT1-Vox	*Both sides containing 1kb homologous fragments of wild-type chromosome V*
bYW0187	pUC19-wtVI-hphNT1-Vox	*Both sides containing 1kb homologous fragments of wild-type chromosome VI*
bYW0188	pUC19-wtIII-KanMX-Vox	*Both sides containing 1kb homologous fragments of wild-type chromosome III*
bYW0189	pUC19-wtIX-KanMX-Vox	*Both sides containing 1kb homologous fragments of wild-type chromosome IX*
bYW0190	pUC19-wtX-KanMX-Vox	*Both sides containing 1kb homologous fragments of wild-type chromosome X*
pYW085	pClB2-CreEBD-CYC1t	*pRS413*
pYW0252	Mat a	*pRS416*
pYW0192	GAL1p-Vika-CYC1t	*pRS415*

Table S5. Primers used in this work.

PCRtag	Len-gth	syn-F (5'-3')	syn-R (5'-3')	wt-F (5'-3')	wt-R (5'-3')
YBL106C_amp1	295	TGATGAACTGCTGATACAACCTCTGGCG	CGGCGATATCGTTATCAGAACCGGTAAG	ACTACTGGAAGATATGCATCCTCGAGCA	TGGTGACATTGTCATTCGCACAGGGAAA
YBL004W_amp7	397	TAGAAGCGACGAACACAGAATGGACAGC	GTGTTTACGTTTTTCTCTGCTACGAGCG	CAGGTCGGATGAACATCGTATGGATTCT	ATGCTTTCTCTTTTCCCTCGATCTCGCA
YBR001C_amp1	388	ACCGGTTAAGCTGTCCAATCTTGGTGAA	TGTCAACCCTAGCACCGGCGAAAGAAGT	CCCTGTCAGAGAATCTAACCTAGGGCTC	CGTTAATCCATCTACTGGTGAAAGGTCC
YBR296C_amp2	478	AACACTGTCGTTGCTGTTTGAAGCAGCT	TGGTGCTATTGCTGCCATCGTCTTCAGC	GACGGAA TCATTAGAATTACTCGCTGCC	AGGCGCGATCGCAGCTATAGTTTTTTCA
YCL064C_amp1	271	ATCTTTGATGGTGTTGCTGCTACCACCG	CAGCTTGGGTACCGCTGTTATTAGCAAC	GTCCTTTATAGTATTAGAGGAGCCGCCA	TTCTCTAGGAACGGCCGTGATCTCTAAT
YCL001W_amp1	295	ACATGCCAAAGAACGATGGGCCGTTCTA	GCTTAGTAGCAAGCTGATAACGGTAGCT	TCACGCTAAGGAGAGGTGGGCTGTATTG	CGACAACAAGAGGGAAATGACAGTGGCT
YCR002C_amp1	217	AGCATTAGAGCTCATGTGGGCGCTGCTT	TTTTCGAGGTCGAAAGACCCGATGGTCT	GGCGTTGCTAGACATATGAGCTGAGGAA	CTTCAGGGGAAGAAAAACTCGTTGGAGC
YCR098C_amp1	334	GCCGCCAAAAGCCAAAGGTAGGTTGGTA	TAGCACCCGAGTCAGCAATGCTGCTTTG	ACCACCGAAGGCTAGTGGCAAATTTGTC	GTCGACTAGAGTTTCCAACGCAGCCCTA
YEL071W_amp1	244	CAGCGGTAGTAACAAACGTCATGATGAC	ATTCAAACGCTCGGTAACGGCAGCGCTA	GTCAGGGTCCAATAAGAGACACGACGAT	GTTTAATCTCTCCGTCACAGCCGCAGAT
YEL001C_amp1	283	AAACAAAGTAGGAGGAGCAGCGATACGA	TGAGGACACTGCCACTTTGGCTTACAAC	GAATAGGGTTGGTGGTGCCGCAATTCTC	GGAGGATACCGCTACCTTAGCCTATAAT
YER001C_amp1	295	GGTTGCTGGCAGAGGCATCGTTTTATCA	CTTGTATGAGCCAAAATCGCCTCTCCAT	AGTAGCCGGTCGTGGTATTGTATTGAGT	TTTATAACTACCGAAGTCACCTCGCCAC
YER188W_amp1	253	TCGTAGAAGCGACGCTTTGGGTGTTACC	GTTCCAACACAACATCTGACCAGGTCTG	AAGACGTTCAGATGCACTTGGAGTGACT	ATTCCAGCATAGCATCTGGCCTGGCCTT
YFL055W_amp2	310	TTACAGCGGTACCGAAAGCGTCGCTCTA	AGTTCGAGAACCGATGTACAAGCTACCG	CTATTCTGGAACGGAATCAGTTGCCTTG	GGTTCTGCTCCCTATATAAAGTGACCCA
YFL001	292	TGACATCCGAA	ATGCCACAAA	CGATATTCGTA	GTGCCATAGG

W_amp1		TCAGCGCCGTT TGTTTG	AAAGCGCTGC CGACCAAG	TATCAGCTGTC TGCCTC	AATGCTGAAC CAACGAGA
YFR001 W_amp1	403	GAGCGCTCAA CGAAAACCAA GCAAGACT	TCGGATGGTTC GAGCAACGCT GCTTTTT	ATCTGCCCAGC GTAAGCCTTCT AAAACC	ACGTATAGTC CTTGCGACAG AAGACTTC
YFR055 W_amp1	268	TTTGGGCAGCA TTAGCCTAGCT TTGCTA	TTTCTTAGCGA CGCTAACTAGG GCAGGG	GCTTGGTTCTA TCTCTTTGGCG CTATTG	CTTTTTGGCA ACAGAGACCA AAGCTGGA
YIL001 W_amp2	484	GTTGTTGTTGC AACGAGGTGC TGTTTGC	TGGGTCAGCA GTGTGTCGAG CCTTA	ACTTCTTCTAC AGAGAGGAGC CGTCTGT	AGGATCGGCG GTATGCCTTG CTTTG
YIR001 C_amp1	382	GTTAGGGCTTT GGTTAGGTGGA TATGGA	CGATAGCCGA AGCATTTTCGT TGGCAAT	ATTTGGAGACT GATTTGGAGG GTACGGT	TGACTCGCGT TCTATCTTTGT CGGTAAC
YIR042 C_amp1	211	ACCGACTTCC AAGCTGCCATT AGCTTCG	CTCTGCTTACT CTGAAGCTGG TCAAAAG	TCCAACTTCTA GTGAACCGTT GGCTTCA	TAGTGCCTATA GTGAGGCAGG CCAAAAA
YJL219 W_amp1	481	TTTAGACGCTA CCGAACCTCC AATCGAT	TAAAGTACCAC GAATTTGCTTT GGGGCG	CCTGGATGCCA CTGAACCACCT ATTGAC	CAGGGTTCCT CTGATTTGTTT GGGAGCA
YJL002 C_amp1	478	GACAAACTTC TCATCGCTACC GCTACTA	TAGCTTCGAGT TCGGTCCATGG GAAGAC	AACGAATTTCT CGTCTGAGCC AGAGGAT	CTCTTTTGAG TTTGGGCCTT GGGAAGAT
YJR001 W_amp2	322	CAGCTTAAGA CGTGCTAACA GCTTCAGA	ACCAGCACCG CCTGACATCAT TTCG	TTCGTTGAGG AGAGCCAATT CGTTTCGT	CCCCGCTCCA CCACTCATCA TTTCT
YJR155 W_amp1	379	CATGGGTGGT GGTCGTTTCCA GTCAAAG	GCTTGGCTTTT TAGTGACGGC AGGATCG	TATGGGAGGC GGGAGATTTC AGAGTAAA	TGAAGGTTTC TTGGTAACAG CTGGGTCA

Table S6. DNA sequences used in this work.

Name	Sequence of homology arms (5' to 3')
vox	AATAGGTCTGAGAACGCCCATTCTCAGACGTATT
KanMX-vox-CEN3-vox	GACATGGAGGCCCAGAATACCCTCCTTGACAGTCTTGACGTGCGCAGCTCAGGGGCATGATGTGACTGTCGCCCGTACATT<br>TAGCCCATACATCCCCATGTATAATCATTTGCATCCATACATTTTGATGGCCGCACGGCGCGAAGCAAAAATTACGGCTCCTC<br>GCTGCAGACCTGCGAGCAGGGAAACGCTCCCCTCACAGACGCGTTGAATTGTCCCCACGCCGCGCCCCTGTAGAGAAATA<br>TAAAAGGTTAGGATTTGCCACTGAGGTTCTTCTTTCATATACTTCCTTTTAAAATCTTGCTAGGATACAGTTCTCACATCACA<br>TCCGAACATAAACAACCATGGGTAAGGAAAAGACTCACGTTTCGAGGCCGCGATTAAATTCCAACATGGATGCTGATTTAT<br>ATGGGTATAAATGGGCTCGCGATAATGTCGGGCAATCAGGTGCGACAATCTATCGATTGTATGGGAAGCCCGATGCGCCAG<br>AGTTGTTTCTGAAACATGGCAAAGGTAGCGTTGCCAATGATGTTACAGATGAGATGGTCAGACTAAACTGGCTGACGGAA<br>TTTATGCCTCTTCCGACCATCAAGCATTTTATCCGTACTCCTGATGATGCATGGTTACTCACCACTGCGATCCCCGGCAAAA<br>CAGCATTCCAGGTATTAGAAGAATATCCTGATTCAGGTGAAAATATTGTTGATGCGCTGGCAGTGTTCCTGCGCCGGTTGCA<br>TTCGATTCCTGTTTGTAATTGTCCTTTTAACAGCGATCGCGTATTTCGTCTCGCTCAGGCGCAATCACGAATGAATAACGGT<br>TTGGTTGATGCGAGTGATTTTGATGACGAGCGTAATGGCTGGCCTGTTGAACAAGTCTGGAAAGAAATGCATAAGCTTTTG<br>CCATTCTCACCGGATTCAGTCGTCACTCATGGTGATTTCTCACTTGATAACCTTATTTTTGACGAGGGGAAATTAATAGGTT<br>GTATTGATGTTGGACGAGTCGGAATCGCAGACCGATACCAGGATCTTGCCATCCTATGGAACTGCCTCGGTGAGTTTTCTC<br>CTTCATTACAGAAACGGCTTTTTCAAAAATATGGTATTGATAATCCTGATATGAATAAATTGCAGTTTCATTTGATGCTCGAT<br>GAGTTTTTCTAATCAGTACTGACAATAAAAAGATTCTTGTTTTCAAGAACTTGTCATTTGTATAGTTTTTTTATATTGTAGTTG<br>TTCTATTTTAATCAAATGTTAGCGTGATTTATATTTTTTTTCGCCTCGACATCATCTGCCCAGATGCGAAGTTAAGTGCGCAG<br>AAAGTAATATCATGCGTCAATCGTATGTGAATGCTGGTCGCTATACTGGTTGCAAATGCTCCGTCGACGGGAATAGGTCTGA<br>GAACGCCCATTCTCAGACGTATTGTACAGTACCTATACATTTCATAAACATGGCATGGCGATCAGCGCCAAACAATATGGAA<br>AATCCACAGAAAGCTATTCATTGAAAAAATAGTACAAATAAGTCACATGATGATATTTGATTTTATTATATTTTTAAAAAAAG<br>TAAAAAATAAAAAGTAGTTTATTTTTAAAAAATAAAATTTAAAATATTAGTGTATTTGATTTCCGAAAGTTAAAAAAGAAAT<br>AGTAAGAAATATATATTTCATTGAATGGATATATGAAACGTTTACTGGTGGAAGTTTTGCTCATATATTATTATTCAATAGAAG<br>TAATAAAGAGTTGCAAATGCTCCGTCGACGGGAATAGGTCTGAGAACGCCCATTCTCAGACGTATT

hphNT1-vox-CEN3-vox	CAGCGACATGGAGGCCCAGAATACCCTCCTTGACAGTCTTGACGTGCGCAGCTCAGGGGCATGATGTGACTGTCGCCCGT<br>ACATTTAGCCCATACATCCCCATGTATAATCATTTGCATCCATACATTTTGATGGCCGCACGGCGCGAAGCAAAAATTACGGC<br>TCCTCGCTGCGGACCTGCGAGCAGGGAAACGCTCCCCTCACAGACGCGTTGAATTGTCCCCACGCCGCGCCCCTGTAGAG<br>AAATATAAAAGGTTAGGATTTGCCACTGAGGTTCTTCTTTCATATACTTCCTTTTAAAATCTTGCTAGGATACAGTTCTCACA<br>TCACATCCGAACATAAACAACCATGGGTAAAAAGCCTGAACTCACCGCGACGTCTGTCGAGAAGTTTCTGATCGAAAAGT<br>TCGACAGCGTCTCCGACCTGATGCAGCTCTCGGAGGGCGAAGAATCTCGTGCTTTCAGCTTCGATGTAGGAGGGCGTGGA<br>TATGTCCTGCGGGTAAATAGCTGCGCCGATGGTTTCTACAAAGATCGTTATGTTTATCGGCACTTTGCATCGGCCGCGCTCC<br>CGATTCCGGAAGTGCTTGACATTGGGGAATTCAGCGAGAGCCTGACCTATTGCATCTCCCGCCGTGCACAGGGTGTCACGT<br>TGCAAGACCTGCCTGAAACCGAACTGCCCGCTGTTCTGCAGCCGGTCGCGGAGGCCATGGATGCGATCGCTGCGGCCGAT<br>CTTAGCCAGACGAGCGGGTTCGGCCCATTCGGACCGCAAGGAATCGGTCAATACACTACATGGCGTGATTTCATATGCGCG<br>ATTGCTGATCCCCATGTGTATCACTGGCAAACTGTGATGGACGACACCGTCAGTGCGTCCGTCGCGCAGGCTCTCGATGAG<br>CTGATGCTTTGGGCCGAGGACTGCCCCGAAGTCCGGCACCTCGTGCACGCGGATTTCGGCTCCAACAATGTCCTGACGGA<br>CAATGGCCGCATAACAGCGGTCATTGACTGGAGCGAGGCGATGTTCGGGGATTCCCAATACGAGGTCGCCAACATCTTCTT<br>CTGGAGGCCGTGGTTGGCTTGTATGGAGCAGCAGACGCGCTACTTCGAGCGGAGGCATCCGGAGCTTGCAGGATCGCCGC<br>GGCTCCGGGCGTATATGCTCCGCATTGGTCTTGACCAACTCTATCAGAGCTTGGTTGACGGCAATTTCGATGATGCAGCTTG<br>GGCGCAGGGTCGATGCGACGCAATCGTCCGATCCGGAGCCGGGACTGTCGGGCGTACACAAATCGCCCGCAGAAGCGCG<br>GCCGTCTGGACCGATGGCTGTGTAGAAGTACTCGCCGATAGTGGAAACCGACGCCCCAGCACTCGTCCGAGGGCAAAGG<br>AATAATCTCGAGTCATGTAATTAGTTATGTCACGCTTACATTCACGCCCTCCCCCCACATCCGCTCTAACCGAAAAGGAAGG<br>AGTTAGACAACCTGAAGTCTAGGTCCCTATTTATTTTTTTATAGTTATGTTAGTATTAAGAACGTTATTTATATTTCAAATTTTT<br>CTTTTTTTTCTGTACAGACGCGTGTACGCATGTAACATTATACTGAAAACCTTGCTTGAGAAGGTTTTGGGACGAATGCTGG<br>TCGCTATACTGGTTGCAAATGCTCCGTCGACGGGAATAGGTCTGAGAACGCCCATTCTCAGACGTATTGTACAGTACCTATA<br>CATTTCATAAACATGGCATGGCGATCAGCGCCAAACAATATGGAAAATCCACAGAAAGCTATTCATTGAAAAAATAGTACA<br>AATAAGTCACATGATGATATTTGATTTTATTATATTTTTAAAAAAAGTAAAAAATAAAAAGTAGTTTATTTTTAAAAAATAAA<br>ATTTAAAATATTAGTGTATTTGATTTCCGAAAGTTAAAAAAGAAATAGTAAGAAATATATATTTCATTGAATGGATATATGAAA<br>CGTTTACTGGTGGAAGTTTTGCTCATATATTATTATTCAATAGAAGTAATAAAGAGTTGCAAATGCTCCGTCGACGGGAATA<br>GGTCTGAGAACGCCCATTCTCAGACGTATT

wtII-hphNT1-vox-CEN3-vox	ATATCAGTGTGTACTCTAACATATCCTTTCTCATGACCGTATTCATGGATTTCGCCAATCAAACCCATCACATCATCCCAAGG
	CCCTTCAATTGTTGTTCCTGCACTGTGTAAAGTGCTCTTTAATGGGCTTTCTCTGATTTTTTTTTCAATGAGTGCAACAAAAT
	CAGAAATACTAGCAGAGTCGGTGCCAATAGGGACCATACACACGTCCGCTAAACAAAAGATCTTGGGCATTTTCTAAACTT
	GTAGTTTATGTGCTTTATAGTTAATGATCACGACGGCAATGACAAAAACTGAATATCTTGATCTCTCCTTTTGACGATGTCAG
	TGAATCCCGGTTAATGATATTCTGTTTTTCACGGGGTGGAAAAGAGGGGAGCTTGAATCATACTAAAAAAGGAATGTCAAA
	GAGCTATATAATTCGAATAAAAAGTAAAAGAAACGAAAGGTTTTATTAAAGGGTTTGGAGCTGTTTTCGGACTGAAAGCCA
	GTAACAAGCTTGTGTAGGAGTTTGTCTGAAAACCATCTTCATTTCAAATAAGAGAGCGAGTAATTTTTAAATAATTTCTTTT
	CTGAACTACTTAAATCGCTATGTACCTTGCGAAAATTATTCTTTAATAGTTTAACTAAAAGTAAAAAGTTAGAAATCATAAAT
	AAAAAATCAAAGAGACAAATCTCGTTGCAAAAGCTTGAAAAATAGTGCTTTACCAACAGGTTGGCAAACCAGAATGCTA
	ACAAAACTTAAAATCCCATTTCCCAAGAGGATCAATCAATCATATTTACCTTGATAGTATAGGATACAAGTGTCTGTGCAGG
	CCTCCGATAACATGTAAAGTAATAAAAGTAGGAGTACTTGCTTTCAGCAGAAAATGAAAAAAAGAACGTTCAAACAGACT
	GAAAATATAGCTATTCAACTGGCACCTACTAATATCAATAATAATAAATTAATCTTGAGCAAATTGATCCTACATAACAGCGA
	CATGGAGGCCCAGAATACCCTCCTTGACAGTCTTGACGTGCGCAGCTCAGGGGCATGATGTGACTGTCGCCCGTACATTTA
	GCCCATACATCCCCATGTATAATCATTTGCATCCATACATTTTGATGGCCGCACGGCGCGAAGCAAAAATTACGGCTCCTCG
	CTGCGGACCTGCGAGCAGGGAAACGCTCCCCTCACAGACGCGTTGAATTGTCCCCACGCCGCGCCCCTGTAGAGAAATAT
	AAAAGGTTAGGATTTGCCACTGAGGTTCTTCTTTCATATACTTCCTTTTAAAATCTTGCTAGGATACAGTTCTCACATCACAT
	CCGAACATAAACAACCATGGGTAAAAAGCCTGAACTCACCGCGACGTCTGTCGAGAAGTTTCTGATCGAAAAGTTCGACA
	GCGTCTCCGACCTGATGCAGCTCTCGGAGGGCGAAGAATCTCGTGCTTTCAGCTTCGATGTAGGAGGGCGTGGATATGTCC
	TGCGGGTAAATAGCTGCGCCGATGGTTTCTACAAAGATCGTTATGTTTATCGGCACTTTGCATCGGCCGCGCTCCCGATTCC
	GGAAGTGCTTGACATTGGGGAATTCAGCGAGAGCCTGACCTATTGCATCTCCCGCCGTGCACAGGGTGTCACGTTGCAAG
	ACCTGCCTGAAACCGAACTGCCCGCTGTTCTGCAGCCGGTCGCGGAGGCCATGGATGCGATCGCTGCGGCCGATCTTAGC
	CAGACGAGCGGGTTCGGCCCATTCGGACCGCAAGGAATCGGTCAATACACTACATGGCGTGATTTCATATGCGCGATTGCT
	GATCCCCATGTGTATCACTGGCAAACTGTGATGGACGACACCGTCAGTGCGTCCGTCGCGCAGGCTCTCGATGAGCTGATG
	CTTTGGGCCGAGGACTGCCCCGAAGTCCGGCACCTCGTGCACGCGGATTTCGGCTCCAACAATGTCCTGACGGACAATGG
	CCGCATAACAGCGGTCATTGACTGGAGCGAGGCGATGTTCGGGGATTCCCAATACGAGGTCGCCAACATCTTCTTCTGGAG
	GCCGTGGTTGGCTTGTATGGAGCAGCAGACGCGCTACTTCGAGCGGAGGCATCCGGAGCTTGCAGGATCGCCGCGGCTCC

	GGGCGTATATGCTCCGCATTGGTCTTGACCAACTCTATCAGAGCTTGGTTGACGGCAATTTCGATGATGCAGCTTGGGCGC<br>AGGGTCGATGCGACGCAATCGTCCGATCCGGAGCCGGGACTGTCGGGCGTACACAAATCGCCCGCAGAAGCGCGGCCGT<br>CTGGACCGATGGCTGTGTAGAAGTACTCGCCGATAGTGGAAACCGACGCCCCAGCACTCGTCCGAGGGCAAAGGAATAAT<br>CTCGAGTCATGTAATTAGTTATGTCACGCTTACATTCACGCCCTCCCCCCACATCCGCTCTAACCGAAAAGGAAGGAGTTA<br>GACAACCTGAAGTCTAGGTCCCTATTTATTTTTTTATAGTTATGTTAGTATTAAGAACGTTATTTATATTTCAAATTTTTCTTTT<br>TTTTCTGTACAGACGCGTGTACGCATGTAACATTATACTGAAAACCTTGCTTGAGAAGGTTTTGGGACGAATGCTGGTCGC<br>TATACTGGTTGCAAATGCTCCGTCGACGGGAATAGGTCTGAGAACGCCCATTCTCAGACGTATTGTACAGTACCTATACATT<br>TCATAAACATGGCATGGCGATCAGCGCCAAACAATATGGAAAATCCACAGAAAGCTATTCATTGAAAAAATAGTACAAATA<br>AGTCACATGATGATATTTGATTTTATTATATTTTTAAAAAAAGTAAAAAATAAAAAGTAGTTTATTTTTAAAAAAATAAAATTTA<br>AAATATTAGTGTATTTGATTTCCGAAAGTTAAAAAAGAAATAGTCTTGAGAAGGTTTTGGGACGAAGAAATATATATTTCAT<br>TGAATGGATATATGAAACGTTTACTGGTGGAAGTTTTGCTCATATATTATTATTCAATAGAAGTAATAAAGAGTTGCAAATGC<br>TCCGTCGACGGGAATAGGTCTGAGAACGCCCATTCTCAGACGTATTAAAAGTTGGTAAAGCAACTTATATTTCAACAAGAA<br>GTATCAATATATTTCTTGCAAAAAATTGTTCACCGAGAATTGACAAAAAAACCTTACGTCGGTGACGACGATATACAGATCC<br>TATATTCTTCATGTATGTAATAGATTCCCATACCTTCTTCGCATTTAAGACACTAATAGAAATGAAAGCTTTCTATTAGTCATT<br>CTTCCTATGCACTAGACCACGCAAAGGTGAACCGGCTTGGTACGAGAATTCTATCACACGGTAATGATAGTGTTCCCGATG<br>TCAAGCAGTCTTATACAGTACTACCCAAGAGCTTAAGAATAAGCAAGAAGATCTGGCAAGCTACAAGGTACTCATAACGAT<br>CTTCAAAGCGTAAGTTATGATCTTATGAAAATCAGACTTTTTTGTCTCAACAAACTCCTAGTTTGAAGATGTTACTGAAATG<br>CCCTTCAAACACTTTTACTTGAAAATCATCGATTACTATTCTAACCTTCACAAATGAGCCTTTACACAACTTGATTAATAATA<br>ATATTTGACCTTACTCTAAATCGTCTCAAATATTACTGAGGCACAATCTCATGGTTATTGATAGAAAGAAGAATATAACATATA<br>TCTTATTTACATATGTTCGTCAATATTATAGGTAATACAATTTTTTCTCAGAGGGTTTCAAACTATTGAAAAATGGCAATGGTG<br>GACTACAAGCAGCGAGAGCCCTTCTTGCATGATTATTCATATATTTCAATCCGAGCAAATAACTTGTGTTCACCCAACCAAA<br>GCCTTCTGTAGCGACGCCCTTAAAATCAGCACCTTGATTACCATATTCCGCATCAACGCGATGCGGATCAGTCCCTCTTGTC<br>ACATCATATTTTTCTACCACCATTCCGTTATAATCAACAAATGATTTTGTAATCATGTACAGCCACCTATAAGCCAATCTTGTA<br>GCTACTTGTTGATAACCATATGCAGATAAACCTTT
wtV-hphNT1-vox-CEN3-vox	TTGCCAGCTATAAAGAAGTGACAGTGAACTTTTACTTGTTTCTTTTGTTTAACCACTAGTATGTAATTGTTTTTGCAGAATTT<br>GAAACTTCACCAAAATCTTCTTAACGTTCAATGACAAAATTACTTACCCGAACTACCGAACTACGAAACATGAATAAATAA

ACTGGAGGCATAAACTCATTTCCAAATTCCCGGAATACATCGTGTAGTCAAGCAGCATACATCACTATCAAAAATCATGCGC
TTGTGATTATCGTATAGTGTATTTATATAATATATATAGATAGCGATAAATGGCAAGATCCTCTATGATAAAAGTCATCATAATG
AAAACAGATGGCGCTTGTCTACTGTTTGCAAGGCTTGAAATCACAGGTTTTTATTATATCGTGGATTTTTGCCATTGCCTAA
ATCAAAAATAATGATCGATTTTTTGTGAATTAGGGAACGGAAGGAAAAAATCAGCAAACAGTCAGCCCTAGCACCATAGG
CCGACAGCAACAAAAACTGTTTTTTTATTCTCAAATATTCATGATATTGATTACACCGTACTTCTTTTCAATGCGTAAACAAC
TAGAGTTGACAAATATTATAAACTAAAAACTATCATATTAATAATATACCTCCTAGCACTTCGTAATGTTTCTGTCATTGAATG
GCGAGAACATAATTTTAAAGAATCAACACCTTAAATTGTTAACCTTAACAAATGAAGTAAATTCAAATATTTACTCTTGAAT
TCTTTTTGCTTTAACTAACACATTGAAATATCAAATTAGTCCACTTTTCTTCCTTGTTGAATGGGTTTCTTCATTAACAGGGG
AACGCTTGCCTACCATCAAGCCCATTCAATGCAGATGTGATTAATTGGTAAACAAAGGGCCAAGCAAAAATACATCTCTCC
TACATGCTACATAAGTCCGAGATATGTTTTATTTAAGAACTATGAATCTGTAAATGACTGATTCAATATTAAACTAAAATTTCT
ATGAAACATCAAATTACAGCGACATGGAGGCCCAGAATACCCTCCTTGACAGTCTTGACGTGCGCAGCTCAGGGGCATGA
TGTGACTGTCGCCCGTACATTTAGCCCATACATCCCCATGTATAATCATTTGCATCCATACATTTTGATGGCCGCACGGCGCG
AAGCAAAAATTACGGCTCCTCGCTGCGGACCTGCGAGCAGGGAAACGCTCCCCTCACAGACGCGTTGAATTGTCCCCACG
CCGCGCCCCTGTAGAGAAATATAAAAGGTTAGGATTTGCCACTGAGGTTCTTCTTTCATATACTTCCTTTTAAAATCTTGCTA
GGATACAGTTCTCACATCACATCCGAACATAAACAACCATGGGTAAAAAGCCTGAACTCACCGCGACGTCTGTCGAGAAG
TTTCTGATCGAAAAGTTCGACAGCGTCTCCGACCTGATGCAGCTCTCGGAGGGCGAAGAATCTCGTGCTTTCAGCTTCGAT
GTAGGAGGGCGTGGATATGTCCTGCGGGTAAATAGCTGCGCCGATGGTTTCTACAAAGATCGTTATGTTTATCGGCACTTTG
CATCGGCCGCGCTCCCGATTCCGGAAGTGCTTGACATTGGGGAATTCAGCGAGAGCCTGACCTATTGCATCTCCCGCCGTG
CACAGGGTGTCACGTTGCAAGACCTGCCTGAAACCGAACTGCCCGCTGTTCTGCAGCCGGTCGCGGAGGCCATGGATGC
GATCGCTGCGGCCGATCTTAGCCAGACGAGCGGGTTCGGCCCATTCGGACCGCAAGGAATCGGTCAATACACTACATGGC
GTGATTTCATATGCGCGATTGCTGATCCCCATGTGTATCACTGGCAAACTGTGATGGACGACACCGTCAGTGCGTCCGTCGC
GCAGGCTCTCGATGAGCTGATGCTTTGGGCCGAGGACTGCCCCGAAGTCCGGCACCTCGTGCACGCGGATTTCGGCTCCA
ACAATGTCCTGACGGACAATGGCCGCATAACAGCGGTCATTGACTGGAGCGAGGCGATGTTCGGGGATTCCCAATACGAG
GTCGCCAACATCTTCTTCTGGAGGCCGTGGTTGGCTTGTATGGAGCAGCAGACGCGCTACTTCGAGCGGAGGCATCCGGA
GCTTGCAGGATCGCCGCGGCTCCGGGCGTATATGCTCCGCATTGGTCTTGACCAACTCTATCAGAGCTTGGTTGACGGCAA
TTTCGATGATGCAGCTTGGGCGCAGGGTCGATGCGACGCAATCGTCCGATCCGGAGCCGGGACTGTCGGGCGTACACAAA

	TCGCCCGCAGAAGCGCGGCCGTCTGGACCGATGGCTGTGTAGAAGTACTCGCCGATAGTGGAAACCGACGCCCCAGCAC<br>TCGTCCGAGGGCAAAGGAATAATCTCGAGTCATGTAATTAGTTATGTCACGCTTACATTCACGCCCTCCCCCCACATCCGCT<br>CTAACCGAAAAGGAAGGAGTTAGACAACCTGAAGTCTAGGTCCCTATTTATTTTTTTATAGTTATGTTAGTATTAAGAACGT<br>TATTTATATTTCAAATTTTTCTTTTTTTTCTGTACAGACGCGTGTACGCATGTAACATTATACTGAAAACCTTGCTTGAGAAG<br>GTTTTGGGACGAATGCTGGTCGCTATACTGGTTGCAAATGCTCCGTCGACGGGAATAGGTCTGAGAACGCCCATTCTCAGA<br>CGTATTGTACAGTACCTATACATTTCATAAACATGGCATGGCGATCAGCGCCAAACAATATGGAAAATCCACAGAAAGCTAT<br>TCATTGAAAAAATAGTACAAATAAGTCACATGATGATATTTGATTTTATTATATTTTTAAAAAAAGTAAAAAATAAAAAGTAG<br>TTTATTTTTAAAAAATAAAATTTAAAATATTAGTGTATTTGATTTCCGAAAGTTAAAAAAGAAATAGTCTTGAGAAGGTTTTG<br>GGACGAAGAAATATATATTTCATTGAATGGATATATGAAACGTTTACTGGTGGAAGTTTTGCTCATATATTATTATTCAATAGA<br>AGTAATAAAGAGTTGCAAATGCTCCGTCGACGGGAATAGGTCTGAGAACGCCCATTCTCAGACGTATTAAAAGTTGGTAA<br>AGCAACTTAAGGTTTCAAGAAAAGGAAAAGAAAAACTAAACGTAGTATAAAGTCTTGTTTAGTGCAAGCCACTGTTGGCG<br>TTTCAACTAAACTATTCCTTAAAAATAAAGTACAATGTTACTGCTAAACAATGTTGAAGGTGAGCTTAAGACAGTAAATCA<br>AGGAAATGCGGTATAATTCCTGCTGACAAAGCCGCACATAAATAGCAGTTGTTAGCTATAAGGAGATTTTTAGCTACGAACA<br>TGGTTGTAATTCTCTTTATCCTGATTACCTTCAAGATTAAGAAAAAAACAACCATGGTAACTACTTTGGCTCAAAGTCAAAC<br>AAAACTTTTACCATTTCACGGTACACGTTTCCGATAAAATATCACTAGCTATTCAATGTCTGTATTCATCGATAAGCCAGAGT<br>ACGACGTACAAAAAATAACGTAAATGCATATCCTGCATCAAGAAAGAACCCTTATGGACGAACAACGAAAGAGATCTAAA<br>TTTAGAAGACAGCAAGACTAACCATTTGATAGCGTAGTAAGGCTTATCTAGAAATGTTGGACAGATAGGAAGAGAAAGTAT<br>AATAATGGTGTATGGTAGATGACTCTATAATCGTGGTCGGAACGTGCAATATATGAGAACGATGAATGCATATACATATGTCG<br>CGACTCCTTTTTTTCCATTATAGCGGCATCGCTTTATTTCGCGTTTTAATTCATTTTCTTTTTTCTTCATTTCTTTCTGCATATG<br>CAAACGCGGCAAGAAGTGCCCACGGATCCGAAGAAAACCTAATACATTGAAGTTTCTCTCCCTGAGATAGGAACTCTCTC<br>TTCTGCGTCAGAAAAAAAAAAAAGTACTCTTTCTGCATATATTGCTCACTACTGACTCACTGATCCTTTTACGAATAGTTGG<br>GCAACACCTATTAAGTTGTAAAACAATCGCGAAAAAAAAAAGAAAACAACCAAGGCATCCCACTTTTCGAACCCTGGCG<br>CTCGGAAGACTTAATCATTGCGA
wtVI-hphNT1-vox-CEN3-vox	TTGGAGGCCATGAATAAGTGTAAACTTGTTCCCTCGATGGTTTTACAAGACTATAAATTTAGCAGATGTGGCAGAACAGAC<br>AAGGGAGTTAGCGCCATGAACCAAGTTATATCGTTAGAAGTCCGTTCCAATTTAACAGATGAGGAACAGCGGGATCCGAC<br>CAACGACAGCAGGGAAATACCCTATGTTCACGTTTTAAATCAATTACTACCCGACGATATTCGTATATCAGCTGTCTGCCTC

AGACCGCCACCTAACTTTGACGCAAGATTCAGCTGTGTTCACCGGCACTATAAGTACATCTTTAATGGAAAAAACCTTAAT
ATTGAAAAAATGTCTAAAGCCGCATCATATTTTGTTGGAGAGAGAGACTTCAGGAATTTTTGTAAGCTTGATGGCTCAAAA
CAAATTACCAATTTTAAACGAACAATAATAAGCTCAAAAATTCTTCCCCTTTCTGAAACTTTCTATTGTTTTGATCTCGTTGG
TTCAGCATTCCTATGGCACCAAGTTCGTTGCATGATGGCCATTCTTTTCTTAGTTGGTCAATCACTTGAAGTGCCGGAAATT
GTCTTGCGTCTGACTGATATTGAGAAAACCCCTCAAAGGCCTGTTTACGAAATGGCAAATGATATACCATTATTGTTATATGA
TTGTAAATTTCCTGAAATGGATTGGCAAGAACCTACTGTAGATGACTATAAGGCAATAAAATTCACGACAGCTACTGAAGC
ATTGACGTTACACTACGAACTTAAGGCCGCAGTATGCAATATTTTTAAAGATGTTTTGCCCACAGCAAATACAAACAACTTC
TCAAAGACAATTATTAATTTGGGCGATGGAAGAGGTAAAGTAGTCGGTACCTATGTGAAACTAGAGGATAGAAGCGTTATG
GAACCTGTCGAAGTTGTTAATGCTAAATACTCCAAGAAAAAGAACAACAAAAATAAGTAAAATAATATATAAACCTGTATA
ATATAACCTTGAAGACTATATTTCTTTTCCAGCGACATGGAGGCCCAGAATACCCTCCTTGACAGTCTTGACGTGCGCAGCT
CAGGGGCATGATGTGACTGTCGCCCGTACATTTAGCCCATACATCCCCATGTATAATCATTTGCATCCATACATTTTGATGGC
CGCACGGCGCGAAGCAAAAATTACGGCTCCTCGCTGCGGACCTGCGAGCAGGGAAACGCTCCCCTCACAGACGCGTTGA
ATTGTCCCCACGCCGCGCCCCTGTAGAGAAATATAAAAGGTTAGGATTTGCCACTGAGGTTCTTCTTTCATATACTTCCTTTT
AAAATCTTGCTAGGATACAGTTCTCACATCACATCCGAACATAAACAACCATGGGTAAAAAGCCTGAACTCACCGCGACGT
CTGTCGAGAAGTTTCTGATCGAAAAGTTCGACAGCGTCTCCGACCTGATGCAGCTCTCGGAGGGCGAAGAATCTCGTGCT
TTCAGCTTCGATGTAGGAGGGCGTGGATATGTCCTGCGGGTAAATAGCTGCGCCGATGGTTTCTACAAAGATCGTTATGTTT
ATCGGCACTTTGCATCGGCCGCGCTCCCGATTCCGGAAGTGCTTGACATTGGGGAATTCAGCGAGAGCCTGACCTATTGCA
TCTCCCGCCGTGCACAGGGTGTCACGTTGCAAGACCTGCCTGAAACCGAACTGCCCGCTGTTCTGCAGCCGGTCGCGGA
GGCCATGGATGCGATCGCTGCGGCCGATCTTAGCCAGACGAGCGGGTTCGGCCCATTCGGACCGCAAGGAATCGGTCAAT
ACACTACATGGCGTGATTTCATATGCGCGATTGCTGATCCCCATGTGTATCACTGGCAAACTGTGATGGACGACACCGTCAG
TGCGTCCGTCGCGCAGGCTCTCGATGAGCTGATGCTTTGGGCCGAGGACTGCCCCGAAGTCCGGCACCTCGTGCACGCGG
ATTTCGGCTCCAACAATGTCCTGACGGACAATGGCCGCATAACAGCGGTCATTGACTGGAGCGAGGCGATGTTCGGGGAT
TCCCAATACGAGGTCGCCAACATCTTCTTCTGGAGGCCGTGGTTGGCTTGTATGGAGCAGCAGACGCGCTACTTCGAGCG
GAGGCATCCGGAGCTTGCAGGATCGCCGCGGCTCCGGGCGTATATGCTCCGCATTGGTCTTGACCAACTCTATCAGAGCTT
GGTTGACGGCAATTTCGATGATGCAGCTTGGGCGCAGGGTCGATGCGACGCAATCGTCCGATCCGGAGCCGGGACTGTCG
GGCGTACACAAATCGCCCGCAGAAGCGCGGCCGTCTGGACCGATGGCTGTGTAGAAGTACTCGCCGATAGTGGAAACCG

	ACGCCCCAGCACTCGTCCGAGGGCAAAGGAATAATCTCGAGTCATGTAATTAGTTATGTCACGCTTACATTCACGCCCTCC<br>CCCCACATCCGCTCTAACCGAAAAGGAAGGAGTTAGACAACCTGAAGTCTAGGTCCCTATTTATTTTTTTATAGTTATGTTA<br>GTATTAAGAACGTTATTTATATTTCAAATTTTTCTTTTTTTTCTGTACAGACGCGTGTACGCATGTAACATTATACTGAAAACC<br>TTGCTTGAGAAGGTTTTGGGACGAATGCTGGTCGCTATACTGGTTGCAAATGCTCCGTCGACGGGAATAGGTCTGAGAAC<br>GCCCATTCTCAGACGTATTGTACAGTACCTATACATTTCATAAACATGGCATGGCGATCAGCGCCAAACAATATGGAAAATC<br>CACAGAAAGCTATTCATTGAAAAAATAGTACAAATAAGTCACATGATGATATTTGATTTTATTATATTTTTAAAAAAAGTAAA<br>AAATAAAAAGTAGTTTATTTTTAAAAAATAAAATTTAAAATATTAGTGTATTTGATTTCCGAAAGTTAAAAAAGAAATAGTC<br>TTGAGAAGGTTTTGGGACGAAGAAATATATATTTCATTGAATGGATATATGAAACGTTTACTGGTGGAAGTTTTGCTCATATA<br>TTATTATTCAATAGAAGTAATAAAGAGTTGCAAATGCTCCGTCGACGGGAATAGGTCTGAGAACGCCCATTCTCAGACGTAT<br>TAAAAGTTGGTAAAGCAACTTATAGGTTGAAAGTTAGAAATTAGTATTATAATAGCAAAAAAAAATTTAAAGTTAGAAATTAG<br>AATTTAAGGCTCTACACACGTTTACGATGATATTGGACGACCGACACGAAAAGACAGTTTTAGGCTTTCTTTTGTCTTGCTC<br>TTGCGTTGAAAGTACGTAATTTCCATTCGATTACTTACTCAACACTTGATATAGAGACCAACTATAAAATAAACTGTTTGGG<br>AAGGAAAGCAGAACTCTTGTAATTACCTCATCTCATTATCAAGAAGCAAAATTTTAACTATGTATTTTGACCTTATTTTTGTA<br>CTCCACTTTTTTTTTTTAGTTTTTTTTAGTTATTTTTTAATTTTGAAGTAACGGCGATGAGGTGAAGTTTTTTTTAAACTGAA<br>AAAGAAGATCGAATGCAAACCATAAATTAGAGATCTAATTGAGGTAAAATTATAAGGTTAGATATCTGTAAATCTATACAATT<br>AAGGAAAGATGGCACCAAAGAAACCTTCTAAGAGACAAAATCTGAGAAGAGAAGTCGCACCAGAGGTGTTTCAAGATTC<br>ACAAGCTAGGAATCAACTAGCGAATGTTCCTCATCTTACCGAAAAATCTGCCCAGCGTAAGCCTTCTAAAACCAAGGTTAA<br>AAAAGAACAGTCTTTGGCTAGACTTTATGGTGCGAAGAAGGACAAGAAGGGGAAATATTCTGAGAAAGACTTGAATATTC<br>CAACACTCAATAGAGCTATCGTTCCGGGTGTTAAAATAAGGAGGGGAAAGAAAGGTAAGAAATTCATTGCTGATAACGAC<br>ACTCTGACTTTAAACCGTTTAATAACAACTATTGGTGACAAGTACGACGATATAGCTGAGAGTAAGCTTGAAAAGGCTAGA<br>AGATTAGAAGAGATACGAGAATTGAAAAGAAAGGAAATTGAAAGAAAGGAAGCGCTTAAACAAGATAAACTAGAAGAA<br>AAAAAAGACGAGATTAAAAAGAAGTCTTCTGTCGCAAGGACTATACGTAGAA
wtIII-KanMX-vox-CEN3-vox	TGCTTATTGAAAGCGTCTTCAATTATATTAAAACTAAAATTGAAGTTTCCAAAAAAGAAAGATAGAACTGATATTAGCAAAT<br>TGTGTGACAAGAAAGAACGGATGACACAGTGGTTAGAAATTTCAATTTTGATGAACTGAGAATAATAATATTATGCTCCCC<br>TGGATTTTATGCGAAGACACTGCTGAAAAAATTTCTGATATATGCAGAGAAGGAGCAAATAAGCCAGTTTTAAGGAACCGA<br>GATAAGCTGTTTTCGCCCATTGTTCTAGTTCTACAAAATATCTACAGGGAATCGATGAGGTTGTAAGAAATCCTGAATACTC

	TATGATAGTGCATAACACTAAAAAGTTGAAAGAATCCCGTATCATGGACGATTTCCTTGAACATTTGAGCAAAGACGATAA<br>CAAAGCATGGTATGGCGCGGAAGAAACCGAGAGAGCTGCAAAATTAGATGCAATAGAAACACTACTTATTACAGATAGTG<br>TACTAAAAAGGAACGACGTGAAAAAACGTGAAAAATACCTAGACCTAATAGAGAATAGTGGAAACAACAATGGCAAAATA<br>TTCGTACTCAGTACTTCAAAAATCACAGTGAGCAACTTGACAAACCAACAGATATAGGCTGTGTCTTAAAATATACCATCC<br>AATACCTTGATGAACTTTTCAAAGATGACTAAAATAAGTGAAATTTCAACATTAACTTCGAATTTTTTTCTTTTTATCTAACA<br>CTTGTCAAACAGAATATAAGGATTACTTGTCTTCTTTGCTACATATTGCTACCACTTCTATTACACAATAGTTTCAATAGCTTG<br>CAGCGTAGCTAAACTCTAAAATTTATCTAAATCACTCATATAAACCGAACCCTTCCCCTTCCGCTTATAGTACAGTACCTATA<br>CATTTCATAAACATGGCATGGCGATCAGCGCCAAACAATATGGAAAATCCACAGAAAGCTATTCATTGAAAAAATAGTACA<br>AATAAGACATGGAGGCCCAGAATACCCTCCTTGACAGTCTTGACGTGCGCAGCTCAGGGGCATGATGTGACTGTCGCCCG<br>TACATTTAGCCCATACATCCCCATGTATAATCATTTGCATCCATACATTTTGATGGCCGCACGGCGCGAAGCAAAAATTACGG<br>CTCCTCGCTGCAGACCTGCGAGCAGGGAAACGCTCCCCTCACAGACGCGTTGAATTGTCCCCACGCCGCGCCCCTGTAGA<br>GAAATATAAAAGGTTAGGATTTGCCACTGAGGTTCTTCTTTCATATACTTCCTTTTAAAATCTTGCTAGGATACAGTTCTCAC<br>ATCACATCCGAACATAAACAACCATGGGTAAGGAAAAGACTCACGTTTCGAGGCCGCGATTAAATTCCAACATGGATGCT<br>GATTTATATGGGTATAAATGGGCTCGCGATAATGTCGGGCAATCAGGTGCGACAATCTATCGATTGTATGGGAAGCCCGATG<br>CGCCAGAGTTGTTTCTGAAACATGGCAAAGGTAGCGTTGCCAATGATGTTACAGATGAGATGGTCAGACTAAACTGGCTG<br>ACGGAATTTATGCCTCTTCCGACCATCAAGCATTTTATCCGTACTCCTGATGATGCATGGTTACTCACCACTGCGATCCCCGG<br>CAAAACAGCATTCCAGGTATTAGAAGAATATCCTGATTCAGGTGAAAATATTGTTGATGCGCTGGCAGTGTTCCTGCGCCG<br>GTTGCATTCGATTCCTGTTTGTAATTGTCCTTTTAACAGCGATCGCGTATTTCGTCTCGCTCAGGCGCAATCACGAATGAATA<br>ACGGTTTGGTTGATGCGAGTGATTTTGATGACGAGCGTAATGGCTGGCCTGTTGAACAAGTCTGGAAAGAAATGCATAAG<br>CTTTTGCCATTCTCACCGGATTCAGTCGTCACTCATGGTGATTTCTCACTTGATAACCTTATTTTTGACGAGGGGAAATTAAT<br>AGGTTGTATTGATGTTGGACGAGTCGGAATCGCAGACCGATACCAGGATCTTGCCATCCTATGGAACTGCCTCGGTGAGTT<br>TTCTCCTTCATTACAGAAACGGCTTTTTCAAAAATATGGTATTGATAATCCTGATATGAATAAATTGCAGTTTCATTTGATGCT<br>CGATGAGTTTTTCTAATCAGTACTGACAATAAAAAGATTCTTGTTTTCAAGAACTTGTCATTTGTATAGTTTTTTTATATTGTA<br>GTTGTTCTATTTTAATCAAATGTTAGCGTGATTTATATTTTTTTTCGCCTCGACATCATCTGCCCAGATGCGAAGTTAAGTGC<br>GCAGAAAGTAATATCATGCGTCAATCGTATGTGAATGCTGGTCGCTATACTGGTTGCAAATGCTCCGTCGACGGGAATAGGT<br>CTGAGAACGCCCATTCTCAGACGTATTGTACAGTACCTATACATTTCATAAACATGGCATGGCGATCAGCGCCAAACAATAT

	GGAAAATCCACAGAAAGCTATTCATTGAAAAAATAGTACAAATAAGTCACATGATGATATTTGATTTTATTATATTTTTAAAAAAAGTAAAAAATAAAAAGTAGTTTATTTTTAAAAAATAAAATTTAAAATATTAGTGTATTTGATTTCCGAAAGTTAAAAAAGAAATAGTAAGAAATATATATTTCATTGAATGGATATATGAAACGTTTACTGGTGGAAGTTTTGCTCATATATTATTATTCAATAGAAGTAATAAAGAGTTGCAAATGCTCCGTCGACGGGAATAGGTCTGAGAACGCCCATTCTCAGACGTATTAAAAGTTGGTAAAGCAACTTAAGAAATAGTAAGAAATATATATTTCATTGAATGGATATATGAAACGTTTACTGGTGGAAGTTTTGCTCATATATTATTATTCAATAGAAGTAATAAAGAAAAAGTTGGTAAAGCAACTTAACAGTAAAAAGGTAATGATTGAAAAAGTTTTTGAACATCTAAGCTATATGTTGATGGGTTTACAATTTTACCATTAGTACTCATGCCTATACTTTTCTGTTCGTCCTTAATGTCCGCGATTTAGAGCAATCATTGAAAGTACTAGATACATTTTAGCCAGAGAGGACTCGTTGACGTAGAATTAAAATTCAAATGAATTTCCGCCCCATTCATATACCCCAAATAACAAACATATTAAAACTTCATAATTATTCAAAATGTGGAGTAGTATAGAAGAGCAGTACCTTCAAAATTGATTTCTTCAGTTTCCCACCCGGGATCCACTTGTCATGCGGTGAGAATCGTATATTGCGTATAATCCGTGTTTCATCACCCACATTATAGTACAAACCTACTGGTGTAACCATTATCATATTCATGACTTGTAGTTAAAAATCTTTCACGATAAATTGAGGAAAAAATCACGTTAAATAGTTTCATATCATTCTTTAGTTAAAGTCTGTGCATAAAAAGAGGATCGGGTGTTGTACTCGTTCTATCAAAATATTGTTATTGTTGCATTATCACACGAATTACAATCAATTTTTTTATCTACAGGCCAGAGTACCTGAAATAATGGAATTAGATTCAAAAAGGATAGCTTAATCGGTCTTTATACTAATTATTTTACGCCCAGAACGAAACATAACGTGCATTAAATCTCACTGTCACAACATTGTATATAATGGTAGCATCGTACTAACTATTGGCATTCATTCAATATTATACAATTATCTTATTAATACTTTCTCAAGTAGTCTTGAAGACCAGCATGTAGGAAGGTGATGATATGCTCCGTAAATAATTGGAAATATTGAGATATAATAACGATGACAAT
wtIXR-KanMX-vox-CEN3-vox	TATAAGTTTACCGAGGTTGCAAGAAGTCAACTTTTAATATTTGTGAACAATTGTATCTTTAGATCTACTGTTGATCTTGCTAATAGTGAACGACGAGTTTTTTCCTTGATGAACTGTCCAGCTTATCCGGATGTTCAACTTATGGTAAAAAACCGAAATGGAGCCATTCGAATATACCCTTGTCACCTGGCTGTTTTGAGCCGCGCAGAATATTTCAAGGTGATGTTCACAAACAACTTCAAGGAGAAAGTTACATATATCAAAGCCAAGCACGTTACAGGAAAGTATAATAGTATCATTCCACAGCTGACATTACCCAATTGTGAGTTTGAGGTTGCCGAAATTATTCTCCGCTACTTATATGCAGATAATACAGACATTCCTTGGATGTATGCCGTCGATGTTTTGTTACTTGCCGATATACTTTTAGAAGACCGCTTAAAAACTATCGCTTCTACAATTATAACACAATCAAAAGAGTTTATTCAGCAATACAATGTTTTTGACGTACTCTATTTATCATGGGAAATAGGAGTGGAACGCTTAGAACAATTTGCAGCTAAATTTATAGCTATACATTTGCAAGAGTTATATAAAGATCCAGAGATTAAAAGGGCAATTATGTTGAGTTCACAACGAATTTCTCTCCGCCAGGAAACTGATACTATTGAACTAGTGGATGACATTAGGTACTATTTGCTCCGAAAATATTCTTTTGAACCGGATGATGTGGAGCTGTTC

GAAAACCAAGACGATTTAGAATACCTAAAACAAGTAGGGTATTTGGAGTACAGAAAAGATATGGGTATGTTAGATAATATC
CTAGCTGATCTGGAACTTGATGTATAATTCCGCATTCAATAACCTTATGTATTTTTACGCGTCACTGCGAACGGATGAATATT
GCCAATGACTGCTGCGCACTCTTAAAAAATATAATGACAATTATAATAAGAAGTATGATACTTTTTCTGTAAAAAATATATAT
ACATATGAGACATGGAGGCCCAGAATACCCTCCTTGACAGTCTTGACGTGCGCAGCTCAGGGGCATGATGTGACTGTCGC
CCGTACATTTAGCCCATACATCCCCATGTATAATCATTTGCATCCATACATTTTGATGGCCGCACGGCGCGAAGCAAAAATTA
CGGCTCCTCGCTGCAGACCTGCGAGCAGGGAAACGCTCCCCTCACAGACGCGTTGAATTGTCCCCACGCCGCGCCCCTGT
AGAGAAATATAAAAGGTTAGGATTTGCCACTGAGGTTCTTCTTTCATATACTTCCTTTTAAAATCTTGCTAGGATACAGTTCT
CACATCACATCCGAACATAAACAACCATGGGTAAGGAAAAGACTCACGTTTCGAGGCCGCGATTAAATTCCAACATGGAT
GCTGATTTATATGGGTATAAATGGGCTCGCGATAATGTCGGGCAATCAGGTGCGACAATCTATCGATTGTATGGGAAGCCCG
ATGCGCCAGAGTTGTTTCTGAAACATGGCAAAGGTAGCGTTGCCAATGATGTTACAGATGAGATGGTCAGACTAAACTGG
CTGACGGAATTTATGCCTCTTCCGACCATCAAGCATTTTATCCGTACTCCTGATGATGCATGGTTACTCACCACTGCGATCCC
CGGCAAAACAGCATTCCAGGTATTAGAAGAATATCCTGATTCAGGTGAAAATATTGTTGATGCGCTGGCAGTGTTCCTGCG
CCGGTTGCATTCGATTCCTGTTTGTAATTGTCCTTTTAACAGCGATCGCGTATTTCGTCTCGCTCAGGCGCAATCACGAATG
AATAACGGTTTGGTTGATGCGAGTGATTTTGATGACGAGCGTAATGGCTGGCCTGTTGAACAAGTCTGGAAAGAAATGCAT
AAGCTTTTGCCATTCTCACCGGATTCAGTCGTCACTCATGGTGATTTCTCACTTGATAACCTTATTTTTGACGAGGGGAAAT
TAATAGGTTGTATTGATGTTGGACGAGTCGGAATCGCAGACCGATACCAGGATCTTGCCATCCTATGGAACTGCCTCGGTG
AGTTTTCTCCTTCATTACAGAAACGGCTTTTTCAAAAATATGGTATTGATAATCCTGATATGAATAAATTGCAGTTTCATTTGA
TGCTCGATGAGTTTTTCTAATCAGTACTGACAATAAAAAGATTCTTGTTTTCAAGAACTTGTCATTTGTATAGTTTTTTTATAT
TGTAGTTGTTCTATTTTAATCAAATGTTAGCGTGATTTATATTTTTTTTCGCCTCGACATCATCTGCCCAGATGCGAAGTTAAG
TGCGCAGAAAGTAATATCATGCGTCAATCGTATGTGAATGCTGGTCGCTATACTGGTTGCAAATGCTCCGTCGACGGGAATA
GGTCTGAGAACGCCCATTCTCAGACGTATTGTACAGTACCTATACATTTCATAAACATGGCATGGCGATCAGCGCCAAACAA
TATGGAAAATCCACAGAAAGCTATTCATTGAAAAAATAGTACAAATAAGTCACATGATGATATTTGATTTTATTATATTTTTAA
AAAAAGTAAAAAATAAAAAGTAGTTTATTTTTAAAAAAATAAAATTTAAAATATTAGTGTATTTGATTTCCGAAAGTTAAAAA
AGAAATAGTAAGAAATATATATTTCATTGAATGGATATATGAAACGTTTACTGGTGGAAGTTTTGCTCATATATTATTATTCAA
TAGAAGTAATAAAGAGTTGCAAATGCTCCGTCGACGGGAATAGGTCTGAGAACGCCCATTCTCAGACGTATTAAAAGTTG
GTAAAGCAACTTTATAATTATTACTTCAGAATAGTTATTTTCACGAATACGAGATACAGGGTAATGAAAACAGGTGTTATCC

	AAAAAAAAAAAATTGATATATTTTAGATACTTTACATACAGAGGCAAGCTTCAGCTGTAGCCAGGTACATAATATTCCCTATAA<br>AGTGATCAATATTTTTTAACGAAAAATTAAAAAAAAAAAATCCTTGAATAAAGGAATGAAAATATTGTAACAAGCGATGACA<br>TTTCTTTTTTTGATTGTAGTTGTGGCATTTTATATTTGCTTTTGCATTTACCCTATTCTTGAAGGTTTGGATAGACAAGCGAGA<br>TAACACCAGTTGTCCTTCAACTTATTCAAAGCAGCAGCTCACTTAATTATTATTAAGTATACACATGAAAGCCTTGTGTTTAT<br>TTCGAATCTTTTTCTTGGGTAGAACTTTTAACGTTGTCAGACGGCATGACAACAGGTTTTTGGGAAGTAGATCTAATATTTT<br>TTGTAGAGTCTTTGGCACTGTTGAAGTGTTTTTTTTGGAAGGTTCTATTCTTGTTATTGTAAGGACTCCTATAATAATTGTTC<br>TTATTATATCCGAAATTTTGATTTGGAGACTGATTTGGAGGGTACGGTGGGTAATAAGGGAAGGTATCGGGATTGGGGTAGG<br>CCATTAAGGGATAATTCCATTGCCATTGTTGGAAATACTGGTTTTGCGAGTTGTAATGCCTATTAAATCCTGGAATGTTAGTT<br>CTTTTTCTGGAGACAGCAATTTTTTTGCCTTTAAGTTCTCCTCCATTTAATTGCAGGGCTTTTTCTCTGTATGCCGGACTTTC<br>AAATTCAATATAGCCGTAGCCTTTTGGTGTTCCCGTATTCCTATCATAAAGAAGTGTTATTCTTTTTATCTGGCCACAGTCTTT<br>GAAATGATCTTCTATTTGCTCTGGGGTGACATCCGGAGTGATGTTACCGACAAAGATAGAACGCGAGTCAGCTTCAAGTTG<br>ATGAGCGTGT
wtX-KanMX-vox-CEN3-vox	AAGTAAAGTCTCAATTGGCCTTATTACTAACTAATAGGTATCTTATAATCACCTAATAAAATAGTATGGCCGTGACATTTAAG<br>GATGGTGTGATACTAGGTGCTGATTCACGTACCACCACTGGTGCGTACATAGCTAACCGTGTGACAGATAAATTAACGAGA<br>GTACATGACAAAATTTGGTGTTGTAGGTCCGGTTCTGCAGCAGACACGCAGGCGATTGCCGACATTGTTCAGTACCATTTG<br>GAATTATATACTTCTCAATATGGTACCCCCTCCACAGAGACTGCTGCCTCGGTGTTCAAAGAATTATGTTACGAAAATAAAG<br>ATAACCTTACTGCTGGTATAATTGTGGCTGGTTATGATGACAAAAACAAAGGGGAAGTATATACTATTCCATTGGGTGGCTC<br>CGTCCATAAGCTGCCTTATGCGATAGCAGGATCTGGCTCTACTTTCATATATGGGTATTGTGATAAAAACTTTAGAGAAAATA<br>TGTCAAAGGAAGAAACCGTAGATTTCATAAAGCATTCGCTATCGCAAGCCATTAAATGGGACGGATCTTCCGGTGGTGTTA<br>TAAGAATGGTTGTTTTGACAGCTGCTGGTGTGGAACGTTTGATATTCTACCCTGATGAATATGAACAACTATAATAGACCCC<br>AAAAGAACAAATTCTGAGTGATTACTTCTCTTTATATTTGTTAACAAATGCCTCTGGATCTACACTGATATAACATATAATAG<br>TCTATAACCTCTTTCTCTTTTTCTCTTCGCTTTACGCTTTTTTCTCTAACTGTTAGAACGACAAAGTATCTCAGAAGGGAATT<br>TCGTAAGCAAGATATTTTACGACTAATCATTTTTTTGTTGTATAAAAACGTATGTGAGATTGATTCACTAATTCTTCGGAGTC<br>CTGGAAGGCTTACCTTATCTATGCATTCGTACAGTTGTGTGCAATGTTAAAAATGGTGACTGTATCTACGTATCTATAAAAAA<br>AGGTTAACTACCGGAAAATCATTTCTTCTCGTAAAGTGTAGACATGGAGGCCCAGAATACCCTCCTTGACAGTCTTGACGT<br>GCGCAGCTCAGGGGCATGATGTGACTGTCGCCCGTACATTTAGCCCATACATCCCCATGTATAATCATTTGCATCCATACATT

	TTGATGGCCGCACGGCGCGAAGCAAAAATTACGGCTCCTCGCTGCAGACCTGCGAGCAGGGAAACGCTCCCCTCACAGA<br>CGCGTTGAATTGTCCCCACGCCGCGCCCCTGTAGAGAAATATAAAAGGTTAGGATTTGCCACTGAGGTTCTTCTTTCATATA<br>CTTCCTTTTAAAATCTTGCTAGGATACAGTTCTCACATCACATCCGAACATAAACAACCATGGGTAAGGAAAAGACTCACG<br>TTTCGAGGCCGCGATTAAATTCCAACATGGATGCTGATTTATATGGGTATAAATGGGCTCGCGATAATGTCGGGCAATCAGG<br>TGCGACAATCTATCGATTGTATGGGAAGCCCGATGCGCCAGAGTTGTTTCTGAAACATGGCAAAGGTAGCGTTGCCAATGA<br>TGTTACAGATGAGATGGTCAGACTAAACTGGCTGACGGAATTTATGCCTCTTCCGACCATCAAGCATTTTATCCGTACTCCT<br>GATGATGCATGGTTACTCACCACTGCGATCCCCGGCAAAACAGCATTCCAGGTATTAGAAGAATATCCTGATTCAGGTGAA<br>AATATTGTTGATGCGCTGGCAGTGTTCCTGCGCCGGTTGCATTCGATTCCTGTTTGTAATTGTCCTTTTAACAGCGATCGCGT<br>ATTTCGTCTCGCTCAGGCGCAATCACGAATGAATAACGGTTTGGTTGATGCGAGTGATTTTGATGACGAGCGTAATGGCTG<br>GCCTGTTGAACAAGTCTGGAAAGAAATGCATAAGCTTTTGCCATTCTCACCGGATTCAGTCGTCACTCATGGTGATTTCTC<br>ACTTGATAACCTTATTTTTGACGAGGGGAAATTAATAGGTTGTATTGATGTTGGACGAGTCGGAATCGCAGACCGATACCAG<br>GATCTTGCCATCCTATGGAACTGCCTCGGTGAGTTTTCTCCTTCATTACAGAAACGGCTTTTTCAAAAATATGGTATTGATAA<br>TCCTGATATGAATAAATTGCAGTTTCATTTGATGCTCGATGAGTTTTTCTAATCAGTACTGACAATAAAAAGATTCTTGTTTT<br>CAAGAACTTGTCATTTGTATAGTTTTTTTATATTGTAGTTGTTCTATTTTAATCAAATGTTAGCGTGATTTATATTTTTTTTCGC<br>CTCGACATCATCTGCCCAGATGCGAAGTTAAGTGCGCAGAAAGTAATATCATGCGTCAATCGTATGTGAATGCTGGTCGCTA<br>TACTGGTTGCAAATGCTCCGTCGACGGGAATAGGTCTGAGAACGCCCATTCTCAGACGTATTGTACAGTACCTATACATTTC<br>ATAAACATGGCATGGCGATCAGCGCCAAACAATATGGAAAATCCACAGAAAGCTATTCATTGAAAAAATAGTACAAATAA<u>G</u><br><u>TCACATGATGATATTTGATTTTATTATATTTTTAAAAAAAGTAAAAAATAAAAAGTAGTTTATTTTTAAAAAAATAAAATTTAAA</u><br><u>ATATTAGTGTATTTGATTTCCGAAAGTTAAAA</u>AAGAAATAGTAAGAAATATATATTTCATTGAATGGATATATGAAACGTTTAC<br>TGGTGGAAGTTTTGCTCATATATTATTATTCAATAGAAGTAATAAAGAGTTGCAAATGCTCCGTCGACGGGAATAGGTCTGA<br>GAACGCCCATTCTCAGACGTATTAAAAGTTGGTAAAGCAACTTTAAGTTACTCTAATATAATCCTATTTAATAATTAGTAGAG<br>TTGTTGAGAACGAGTATAATATTATTTTTTTTTTATTTTTTTGTAGGTTCTTTGGCCATCCTAAATAGAATAAGCATCCACAAC<br>AACTAAACTGGTATTTTGTTATACACAACGCGTCTTGGTTAGGCATCTGTTTTGTTTTAGCTATTGTTCATCGCGTTTTTTTTT<br>TTCTTATCTCTTTTACCGAGGGGCGAAAATAGCGATAGATCGAGAATATAAATAAATAAAATATTGTTGTGTTGTGTAGTTCT<br>AAAGAAAAATTTTACAGTGAAAAGGTAACACCGGGGAGTCATTACGTAGACTTACGTATTCTGTATAACTGATTCCGAGAC<br>GCAAATGCCTGAGCAAGAACCATTGAGTCCCAATGGCCGTAAGCGCTCTGAGGTTCACTATATCTCAATCCCATTGAACAG

	AGGTTCCGCATTCTCACCAGATGATTCTGTATCACAGTTTCAGTCTGACGGGTTCATGACACGTAGGCAATCCATATTGGAC<br>CATCCTGTGGGCTCATTTAAGGGAGTTAATTCTTTAAGTCGATTTGCGACTTCGTTGAGGAGAGCCAATTCGTTTCGTAATA<br>TCGAGCTAAATGCGGATAATGAAAGATCCTTTTTCAAAGAGAGTAACGATGAAACCTACGATCCGGATACTTTAGCACCAG<br>CTTTGGACGGTAGAAGATTATCAGTAACTTTAAATAATGCTGGTCGCCCGCGCATTACTAATTTGGCTAACAACGATAGAGT<br>TAGTACAGCCAGCATGGCTATTCACGATGATGATTATGGGTCCATCCAAAATTCAACAATTGGGGATTCTGGGTCAATATTA<br>CGCCCTACTGCCTCCTTAACAGAAATGATGAGTGGTGGAGCGGGGAGAAGGTTTACAAATAATGACATGGATTCAATTGTA<br>GTGAAGAGAGTGGAGGGTGTAGATGGTAAGGTAGTAACTCTTCTTGCTGGTCAGTCGACCGCTCCACAGACCATATTCAA<br>CTCA
GAL1p-Vika-CYC1t	TCGCGCGTTTCGGTGATGACGGTGAAAACCTCTGACACATGCAGCTCCCGGAGACGGTCACAGCTTGTCTGTAAGCGGAT<br>GCCGGGAGCAGACAAGCCCGTCAGGGCGCGTCAGCGGGTGTTGGCGGGTGTCGGGGCTGGCTTAACTATGCGGCATCAG<br>AGCAGATTGTACTGAGAGTGCACCATATCGACTACGTCGTAAGGCCGTTTCTGACAGAGTAAAATTCTTGAGGGAACTTTC<br>ACCATTATGGGAAATGGTTCAAGAAGGTATTGACTTAAACTCCATCAAATGGTCAGGTCATTGAGTGTTTTTTATTTGTTGT<br>ATTTTTTTTTTTTTAGAGAAAATCCTCCAATATCAAATTAGGAATCGTAGTTTCATGATTTTCTGTTACACCTAACTTTTTGTG<br>TGGTGCCCTCCTCCTTGTCAATATTAATGTTAAAGTGCAATTCTTTTTCCTTATCACGTTGAGCCATTAGTATCAATTTGCTTA<br>CCTGTATTCCTTTACTATCCTCCTTTTTCTCCTTCTTGATAAATGTATGTAGATTGCGTATATAGTTTCGTCTACCCTATGAACA<br>TATTCCATTTTGTAATTTCGTGTCGTTTCTATTATGAATTTCATTTATAAAGTTTATGTACAAATATCATAAAAAAAGAGAATCT<br>TTTTAAGCAAGGATTTTCTTAACTTCTTCGGCGACAGCATCACCGACTTCGGTGGTACTGTTGGAACCACCTAAATCACCA<br>GTTCTGATACCTGCATCCAAAACCTTTTTAACTGCATCTTCAATGGCCTTACCTTCTTCAGGCAAGTTCAATGACAATTTCA<br>ACATCATTGCAGCAGACAAGATAGTGGCGATAGGGTCAACCTTATTCTTTGGCAAATCTGGAGCAGAACCGTGGCATGGTT<br>CGTACAAACCAAATGCGGTGTTCTTGTCTGGCAAAGAGGCCAAGGACGCAGATGGCAACAAACCCAAGGAACCTGGGAT<br>AACGGAGGCTTCATCGGAGATGATATCACCAAACATGTTGCTGGTGATTATAATACCATTTAGGTGGGTTGGGTTCTTAACT<br>AGGATCATGGCGGCAGAATCAATCAATTGATGTTGAACCTTCAATGTAGGGAATTCGTTCTTGATGGTTTCCTCCACAGTTT<br>TTCTCCATAATCTTGAAGAGGCCAAAACATTAGCTTTATCCAAGGACCAAATAGGCAATGGTGGCTCATGTTGTAGGGCCA<br>TGAAAGCGGCCATTCTTGTGATTCTTTGCACTTCTGGAACGGTGTATTGTTCACTATCCCAAGCGACACCATCACCATCGTC<br>TTCCTTTCTCTTACCAAAGTAAATACCTCCCACTAATTCTCTGACAACAACGAAGTCAGTACCTTTAGCAAATTGTGGCTTG<br>ATTGGAGATAAGTCTAAAAGAGAGTCGGATGCAAAGTTACATGGTCTTAAGTTGGCGTACAATTGAAGTTCTTTACGGATT

	TTTAGTAAACCTTGTTCAGGTCTAACACTACCGGTACCCCATTTAGGACCACCCACAGCACCTAACAAAACGGCATCAACC<br>TTCTTGGAGGCTTCCAGCGCCTCATCTGGAAGTGGGACACCTGTAGCATCGATAGCAGCACCACCAATTAAATGATTTTCG<br>AAATCGAACTTGACATTGGAACGAACATCAGAAATAGCTTTAAGAACCTTAATGGCTTCGGCTGTGATTTCTTGACCAACG<br>TGGTCACCTGGCAAAACGACGATCTTCTTAGGGGCAGACATAGGGGCAGACATTAGAATGGTATATCCTTGAAATATATATA<br>TATATTGCTGAAATGTAAAAGGTAAGAAAAGTTAGAAAGTAAGACGATTGCTAACCACCTATTGGAAAAAACAATAGGTCC<br>TTAAATAATATTGTCAACTTCAAGTATTGTGATGCAAGCATTTAGTCATGAACGCTTCTCTATTCTATATGAAAAGCCGGTTC<br>CGGCCTCTCACCTTTCCTTTTTCTCCCAATTTTTCAGTTGAAAAAGGTATATGCGTCAGGCGACCTCTGAAATTAACAAAAA<br>ATTTCCAGTCATCGAATTTGATTCTGTGCGATAGCGCCCCTGTGTGTTCTCGTTATGTTGAGGAAAAAAATAATGGTTGCTA<br>AGAGATTCGAACTCTTGCATCTTACGATACCTGAGTATTCCCACAGTTAACTGCGGTCAAGATATTTCTTGAATCAGGCGCC<br>TTAGACCGCTCGGCCAAACAACCAATTACTTGTTGAGAAATAGAGTATAATTATCCTATAAATATAACGTTTTTGAACACAC<br>ATGAACAAGGAAGTACAGGACAATTGATTTTGAAGAGAATGTGGATTTTGATGTAATTGTTGGGATTCCATTTTTAATAAGG<br>CAATAATATTAGGTATGTGGATATACTAGAAGTTCTCCTCGACCGTCGATATGCGGTGTGAAATACCGCACAGATGCGTAAG<br>GAGAAAATACCGCATCAGGAAATTGTAAACGTTAATATTTTGTTAAAATTCGCGTTAAATTTTTGTTAAATCAGCTCATTTTT<br>TAACCAATAGGCCGAAATCGGCAAAATCCCTTATAAATCAAAAGAATAGACCGAGATAGGGTTGAGTGTTGTTCCAGTTTG<br>GAACAAGAGTCCACTATTAAAGAACGTGGACTCCAACGTCAAAGGGCGAAAAACCGTCTATCAGGGCGATGGCCCACTA<br>CGTGAACCATCACCCTAATCAAGTTTTTTGGGGTCGAGGTGCCGTAAAGCACTAAATCGGAACCCTAAAGGGAGCCCCCG<br>ATTTAGAGCTTGACGGGGAAAGCCGGCGAACGTGGCGAGAAAGGAAGGGAAGAAAGCGAAAGGAGCGGGCGCTAGGG<br>CGCTGGCAAGTGTAGCGGTCACGCTGCGCGTAACCACCACACCCGCCGCGCTTAATGCGCCGCTACAGGGCGCGTCGCGC<br>CATTCGCCATTCAGGCTGCGCAACTGTTGGGAAGGGCGATCGGTGCGGGCCTCTTCGCTATTACGCCAGCTGGCGAAAGG<br>GGGATGTGCTGCAAGGCGATTAAGTTGGGTAACGCCAGGGTTTTCCCAGTCACGACGTTGTAAAACGACGGCCAGTGAGC<br>GCGCGTAATACGACTCACTATAGGGCGAATTGGGTACCGGGCCCCCCCTCGAGGTCGACGGTATCGATAAGCTTGATATCG<br>AATTCCGGATTAGAAGCCGCCGAGCGGGTGACAGCCCTCCGAAGGAAGACTCTCCTCCGTGCGTCCTCGTCTTCACCGGT<br>CGCGTTCCTGAAACGCAGATGTGCCTCGCGCCGCACTGCTCCGAACAATAAAGATTCTACAATACTAGCTTTTATGGTTATG<br>AAGAGGAAAAATTGGCAGTAACCTGGCCCCACAAACCTTCAAATGAACGAATCAAATTAACAACCATAGGATGATAATGC<br>GATTAGTTTTTTAGCCTTATTTCTGGGGTAATTAATCAGCGAAGCGATGATTTTTGATCTATTAACAGATATATAAATGCAAAA<br>ACTGCATAACCACTTTAACTAATACTTTCAACATTTTCGGTTTGTATTACTTCTTATTCAAATGTAATAAAAGTATCAACAAA

AAATTGTTAATATACCTCTATACTTTAACGTCAAGGAGAAAAAACCCCGGATCGGACTACTAGCAGCTGTAATACGACTCAC
TATAGGGAATATTAAGCTTATGACTGATTTGACTCCTTTCCCACCATTAGAGCACTTGGAGCCAGACGAGTTTGCAGACTTG
GTCAGGAAGGCTATTAAGAGGGACCCACAAGCAGGTGCTCACCCAGCAATTCAATCTGCTATTTCTCACTTTCAGGATGAG
TTCGTCAGGAGGCAAGGTGAATGGCAACCAGCTACTTTGCAAAGATTGAGAAATGCTTGGAACGTTTTCGTCAGATGGTG
CACACACCAGGGTATTCCAGCTTTGCCAGCAAGGCACCAAGATGTCGAAAGGTACTTGATTGAGAGAAGAAATGAATTGC
ATAGAAATACTTTGAAAGTTCATTTATGGGCTATTGGTAAAACACATGTCATTTCTGGTTTGCCAAACCCTTGCGCTCATAG
GTACGTTAAGGCTCAAATGGCACAAATTACTCACCAGAAGGTCAGGGAAAGGGAAAGGATTGAGCAGGCTCCTGCTTTTA
GGGAGTCTGACTTGGACAGGTTGACAGAGTTGTGGTCTGCTACTAGGTCTGTCACTCAGCAAAGAGATTTAATGATTGTTT
CTTTGGCTTATGAGACATTATTGAGAAAGAACAATTTGGAACAAATGAAAGTTGGTGATATTGAATTTTGTCAAGATGGTTC
AGCTTTGATTACTATTCCATTCTCTAAAACAAACCACTCTGGTAGAGATGACGTCAGGTGGATTTCTCCACAGGTCGCTAAC
CAGGTTCATGCTTACTTGCAATTACCAAATATTGATGCTGATCCTCAATGTTTTTTATTGCAAAGAGTTAAAAGATCTGGTAA
GGCGCTGAATCCAGAATCACACAATACTTTGAATGGTCATCATCCAGTTTCTGAAAAATTGATTAGCAGGGTCTTCGAAAG
GGCATGGAGAGCTTTGAACCACGAGACTGGTCCAAGGTATACTGGTCACTCTGCTAGAGTCGGTGCTGCTCAAGACTTGT
TGCAGGAGGGTTACTCAACTTTGCAGGTCATGCAGGCTGGTGGATGGTCTTCAGAGAAGATGGTCTTGAGGTACGGTAGG
CACTTGCATGCACACACATCAGCTATGGCTCAGAAGAGGAGACAAAGATAATCTAGAGGGCCGCATCATGTAATTAGTTAT
GTCACGCTTACATTCACGCCCTCCCCCCACATCCGCTCTAACCGAAAAGGAAGGAGTTAGACAACCTGAAGTCTAGGTCC
CTATTTATTTTTTTATAGTTATGTTAGTATTAAGAACGTTATTTATATTTCAAATTTTTCTTTTTTTTCTGTACAGACGCGTGTAC
GCATGTAACATTATACTGAAAACCTTGCTTGAGAAGGTTTTGGGACGCTCGAAGGCTTTAATTTGCgaattcCTGCAGCCCGG
GGGATCCACTAGTTCTAGAGCGGCCGCCACCGCGGTGGAGCTCCAGCTTTTGTTCCCTTTAGTGAGGGTTAATTGCGCGCT
TGGCGTAATCATGGTCATAGCTGTTTCCTGTGTGAAATTGTTATCCGCTCACAATTCCACACAACATAGGAGCCGGAAGCAT
AAAGTGTAAAGCCTGGGGTGCCTAATGAGTGAGGTAACTCACATTAATTGCGTTGCGCTCACTGCCCGCTTTCCAGTCGGG
AAACCTGTCGTGCCAGCTGCATTAATGAATCGGCCAACGCGCGGGGAGAGGCGGTTTGCGTATTGGGCGCTCTTCCGCTT
CCTCGCTCACTGACTCGCTGCGCTCGGTCGTTCGGCTGCGGCGAGCGGTATCAGCTCACTCAAAGGCGGTAATACGGTTAT
CCACAGAATCAGGGGATAACGCAGGAAAGAACATGTGAGCAAAAGGCCAGCAAAAGGCCAGGAACCGTAAAAAGGCCG
CGTTGCTGGCGTTTTTCCATAGGCTCCGCCCCCCTGACGAGCATCACAAAAATCGACGCTCAAGTCAGAGGTGGCGAAAC
CCGACAGGACTATAAAGATACCAGGCGTTTCCCCCTGGAAGCTCCCTCGTGCGCTCTCCTGTTCCGACCCTGCCGCTTACC

GGATACCTGTCCGCCTTTCTCCCTTCGGGAAGCGTGGCGCTTTCTCATAGCTCACGCTGTAGGTATCTCAGTTCGGTGTAGG
TCGTTCGCTCCAAGCTGGGCTGTGTGCACGAACCCCCCGTTCAGCCCGACCGCTGCGCCTTATCCGGTAACTATCGTCTTG
AGTCCAACCCGGTAAGACACGACTTATCGCCACTGGCAGCAGCCACTGGTAACAGGATTAGCAGAGCGAGGTATGTAGGC
GGTGCTACAGAGTTCTTGAAGTGGTGGCCTAACTACGGCTACACTAGAAGGACAGTATTTGGTATCTGCGCTCTGCTGAAG
CCAGTTACCTTCGGAAAAAGAGTTGGTAGCTCTTGATCCGGCAAACAAACCACCGCTGGTAGCGGTGGTTTTTTTGTTTGC
AAGCAGCAGATTACGCGCAGAAAAAAAGGATCTCAAGAAGATCCTTTGATCTTTTCTACGGGGTCTGACGCTCAGTGGAA
CGAAAACTCACGTTAAGGGATTTTGGTCATGAGATTATCAAAAAGGATCTTCACCTAGATCCTTTTAAATTAAAAATGAAGT
TTTAAATCAATCTAAAGTATATATGAGTAAACTTGGTCTGACAGTTACCAATGCTTAATCAGTGAGGCACCTATCTCAGCGAT
CTGTCTATTTCGTTCATCCATAGTTGCCTGACTCCCCGTCGTGTAGATAACTACGATACGGGAGGGCTTACCATCTGGCCCC
AGTGCTGCAATGATACCGCGAGACCCACGCTCACCGGCTCCAGATTTATCAGCAATAAACCAGCCAGCCGGAAGGGCCGA
GCGCAGAAGTGGTCCTGCAACTTTATCCGCCTCCATCCAGTCTATTAATTGTTGCCGGGAAGCTAGAGTAAGTAGTTCGCC
AGTTAATAGTTTGCGCAACGTTGTTGCCATTGCTACAGGCATCGTGGTGTCACGCTCGTCGTTTGGTATGGCTTCATTCAGC
TCCGGTTCCCAACGATCAAGGCGAGTTACATGATCCCCCATGTTGTGCAAAAAAGCGGTTAGCTCCTTCGGTCCTCCGATC
GTTGTCAGAAGTAAGTTGGCCGCAGTGTTATCACTCATGGTTATGGCAGCACTGCATAATTCTCTTACTGTCATGCCATCCG
TAAGATGCTTTTCTGTGACTGGTGAGTACTCAACCAAGTCATTCTGAGAATAGTGTATGCGGCGACCGAGTTGCTCTTGCC
CGGCGTCAATACGGGATAATACCGCGCCACATAGCAGAACTTTAAAAGTGCTCATCATTGGAAAACGTTCTTCGGGGCGAA
AACTCTCAAGGATCTTACCGCTGTTGAGATCCAGTTCGATGTAACCCACTCGTGCACCCAACTGATCTTCAGCATCTTTTAC
TTTCACCAGCGTTTCTGGGTGAGCAAAAACAGGAAGGCAAAATGCCGCAAAAAAGGGAATAAGGGCGACACGGAAATGT
TGAATACTCATACTCTTCCTTTTTCAATATTATTGAAGCATTTATCAGGGTTATTGTCTCATGAGCGGATACATATTTGAATGTA
TTTAGAAAAATAAACAAATAGGGGTTCCGCGCACATTTCCCCGAAAAGTGCCACCTGGGTCCTTTTCATCACGTGCTATAA
AAATAATTATAATTTAAATTTTTTAATATAAATATATAAATTAAAAATAGAAAGTAAAAAAAGAAATTAAAGAAAAAATAGTT
TTTGTTTTCCGAAGATGTAAAAGACTCTAGGGGGATCGCCAACAAATACTACCTTTTATCTTGCTCTTCCTGCTCTCAGGTA
TTAATGCCGAATTGTTTCATCTTGTCTGTGTAGAAGACCACACACGAAAATCCTGTGATTTTACATTTTACTTATCGTTAATC
GAATGTATATCTATTTAATCTGCTTTTCTTGTCTAATAAATATATATGTAAAGTACGCTTTTTGTTGAAATTTTTTAAACCTTTG
TTTATTTTTTTTTCTTCATTCCGTAACTCTTCTACCTTCTTTATTTACTTTCTAAAATCCAAATACAAAACATAAAAATAAATA
AACACAGAGTAAATTCCCAAATTATTCCATCATTAAAAGATACGAGGCGCGTGTAAGTTACAGGCAAGCGATCCGTCCTAA

GAAACCATTATTATCATGACATTAACCTATAAAAATAGGCGTATCACGAGGCCCTTTCGTC

References:

1. Xie Z, Li B, Mitchell LA *et al*. “Perfect” designer chromosome V and behavior of a ring derivative. *Science* 2017; **355**: f4704.
2. Shen Y, Wang Y, Chen T *et al*. Deep functional analysis of synII, a 770-kilobase synthetic yeast chromosome. *Science* 2017; **355**: f4791.
3. Mercy G, Mozziconacci J, Scolari VF *et al*. 3D organization of synthetic and scrambled chromosomes. *Science* 2017; **355**: f4597.
4. Mitchell LA, Wang A, Stracquadanio G *et al*. Synthesis, debugging, and effects of synthetic chromosome consolidation: SynVI and beyond. *Science* 2017; **355**: f4831.
5. Annaluru N, Muller H, Mitchell LA *et al*. Total synthesis of a functional designer eukaryotic chromosome. *Science* 2014; **344**: 55-58.
6. Dymond JS, Richardson SM, Coombes CE *et al*. Synthetic chromosome arms function in yeast and generate phenotypic diversity by design. *Nature* 2011; **477**: 471-476.
7. Gibson DG, Young L, Chuang R *et al*. Enzymatic assembly of DNA molecules up to several hundred kilobases. *Nat Methods* 2009; **6**: 343-345.
8. Gietz RD, Schiestl RHW, A. R. Woods R. Studies on the transformation of intact yeast cells by the LiAc/SS-DNA/PEG procedure. *Yeast* 1995; **11**: 355-360.
9. Richardson SM, Mitchell LA, Stracquadanio G *et al*. Design of a synthetic yeast genome. *Science* 2017; **355**: 1040-1044.
10. Lin Q, Qi H, Wu Y *et al*. Robust orthogonal recombination system for versatile genomic elements rearrangement in yeast *Saccharomyces cerevisiae*. *Sci Rep-UK* 2015; **5**: 15249.
11. Flatters M, Maxfield R, Dawson D. The eects of a ring chromosome on the meiotic segregation of other chromosomes in *Saccharomyces cerevisiae*. *Mol Gen Genet* 1995; **249**: 309-316.
12. Haber JE, Thorburn PC, Rogers D. Meiotic and mitotic behavior of dicentric chromosomes in *Saccharomyces cerevisiae*. *Genetics* 1984; **106**: 185-205.
13. Shen MJ, Wu Y, Yang K *et al*. Heterozygous diploid and interspecies SCRaMbLEing. *Nat Commun* 2018; **9**: 1-8.
14. Shen Y, Stracquadanio G, Wang Y *et al*. SCRaMbLE generates designed combinatorial stochastic diversity in synthetic chromosomes. *Genome Res.* 2016; **26**: 36-49.
15. Kim D, Langmead B, Salzberg SL. HISAT: A fast spliced aligner with low memory requirements. *Nat Methods* 2015; **12**: 357-360.
16. Li B, Dewey CN. RSEM: Accurate transcript quantification from RNA-Seq data with or without a reference genome. *Bmc Bioinformatics* 2011; **12**: 323.
17. Trapnell C, Williams BA, Pertea G *et al*. Transcript assembly and quantification by RNA-Seq reveals unannotated transcripts and isoform switching during cell differentiation. *Nat Biotechnol* 2010; **28**: 511-515.
18. Zhang Y, Liu T, Meyer CA *et al*. Model-based analysis of ChIP-Seq (MACS). *Genome Biol* 2008; **9**: R137.
19. Schep AN, Buenrostro JD, Denny SK *et al*. Structured nucleosome fingerprints enable high-resolution mapping of chromatin architecture within regulatory regions. *Genome Res* 2015; **25**: 1757-1770.
20. Lieberman-Aiden E, van Berkum NL, Williams L *et al*. Comprehensive mapping of long-gange

interactions reveals folding principles of the human genome. *Science* 2009; **326**: 289-293.
21. Varoquaux N, Ay F, Noble WS *et al*. A statistical approach for inferring the 3D structure of the genome. *Bioinformatics* 2014; **30**: i26-i33.
